# Targeting pathogen metabolism without collateral damage to the host

**DOI:** 10.1038/srep40406

**Published:** 2017-01-13

**Authors:** Jurgen R. Haanstra, Albert Gerding, Amalia M. Dolga, Freek J. H. Sorgdrager, Manon Buist-Homan, François du Toit, Klaas Nico Faber, Hermann-Georg Holzhütter, Balázs Szöör, Keith R. Matthews, Jacky L. Snoep, Hans V. Westerhoff, Barbara M. Bakker

**Affiliations:** 1 University of Groningen, University Medical Center Groningen, Department of Pediatrics and Systems Biology Centre for Energy Metabolism and Ageing, Center for Liver, Digestive and Metabolic Diseases, Groningen, The Netherlands; 2 Department of Molecular Cell Physiology, Faculty of Earth and Life Sciences, VU University, Amsterdam, The Netherlands; 3 University of Groningen, Department of Molecular Pharmacology, Groningen, The Netherlands; 4 University of Groningen, University Medical Center Groningen, European Research Institute for the Biology of Ageing, Groningen, The Netherlands; 5 University of Groningen, University Medical Center Groningen, Department of Hepatology and Gastroenterology, and Department of Laboratory Medicine, Groningen, The Netherlands; 6 Department of Biochemistry, Stellenbosch University, South Africa; 7 Charité - Universitätsmedizin Berlin, Institut für Biochemie, Berlin, Germany; 8 Centre for Immunity, Infection and Evolution, Institute for Immunology and Infection Research, School of Biological Sciences, University of Edinburgh, UK; 9 Manchester Centre for Integrative Systems Biology, School of Chemical Engineering and Analytical Science, University of Manchester, Manchester, UK; 10 Swammerdam Institute for Life Sciences, Faculty of Science, University of Amsterdam, Amsterdam, The Netherlands

## Abstract

The development of drugs that can inactivate disease-causing cells (e.g. cancer cells or parasites) without causing collateral damage to healthy or to host cells is complicated by the fact that many proteins are very similar between organisms. Nevertheless, due to subtle, quantitative differences between the biochemical reaction networks of target cell and host, a drug can limit the flux of the same essential process in one organism more than in another. We identified precise criteria for this ‘network-based’ drug selectivity, which can serve as an alternative or additive to structural differences. We combined computational and experimental approaches to compare energy metabolism in the causative agent of sleeping sickness, *Trypanosoma brucei*, with that of human erythrocytes, and identified glucose transport and glyceraldehyde-3-phosphate dehydrogenase as the most selective antiparasitic targets. Computational predictions were validated experimentally in a novel parasite-erythrocytes co-culture system. Glucose-transport inhibitors killed trypanosomes without killing erythrocytes, neurons or liver cells.

In the treatment of both cancer and infectious diseases, the major challenge is to design selective drugs that target the cancer cell or pathogen without harming the patient. Indeed, two main reasons for the failure of candidate drugs at a late stage are lack of a therapeutic effect and problems with drug safety^1^,^2^,^3^. Classically, drug-design strategies are aimed at a molecular target that is both essential and unique to the diseased cell. Often such choices are limited, however. For instance, cancer cells originate from healthy cells and their genomes are virtually identical to those of healthy cells. Therefore, unique cancer proteins are rare. Analogously, eukaryotic pathogens tend to be biochemically similar to some cells of the host. In such cases we may need to drop the criterion of uniqueness and seriously consider drug targets that are part of pathways that also occur in the host and that may even be essential there.

Pathogen proliferation and survival depend on metabolism for the supply of free energy and molecular building blocks. This makes metabolic enzymes attractive drug targets. Of course, a putative target enzyme should be essential for pathogen survival. Researchers assess this experimentally through gene knockouts or computationally using genome-scale metabolic models^4^. Comparative computational analysis of constraint-based models allows the identification of targets that are essential for the pathogen but not for the host^5^. At safe doses, however, inhibitors rarely cause 100% inhibition. The resulting residual enzyme activity makes chemical inhibition essentially different from a gene knockout, as is implicitly acknowledged by the use of IC_50_ rather than IC_100_ values for drug efficacy. This means that when identifying effective drug targets we must consider additional criteria besides essentiality.

The overall effect of an enzyme inhibitor on cell function depends on multiple factors. Classically, the focus is on the efficiency of inhibitor uptake by the cell and on the binding affinity of inhibitors for their target enzyme. These factors may be optimized by altering the chemical structure of inhibitors, resulting in *structure-based* drug selectivity (see e.g. ref. 6). *In vivo*, however, two other important factors must be taken into consideration. First, the direct effect of the inhibitor on enzyme activity also depends on concentrations of intracellular metabolites that act on the target enzyme. Thus, competitive inhibitors may be competed away by rising substrate concentrations, while uncompetitive inhibitors become more effective at high substrate concentrations^7^. Second, the control exerted on cell function (i.e. the extent to which an enzyme is rate-limiting) differs considerably between putative target enzymes, and even between sequential and essential enzymes in a single metabolic pathway (e.g. ref. 8). Some very important enzymes are present in excess and need to be inhibited almost completely before cell function is affected, while others are present in limiting amounts^9^. These two factors – interacting metabolite concentrations and the target enzyme’s degree of control over cell function – depend on the kinetic properties of the metabolic network as a whole and are important for the efficacy and selectivity of drugs *in vivo*. These network properties should therefore be included when ranking putative drug targets. They constitute the basis for *network-based* drug selectivity.

In this study, we developed the concept of network-based drug selectivity and applied it to glycolysis in *Trypanosoma brucei* – the parasite that causes African sleeping sickness *–* and glycolysis in the erythrocytes of its human host. We aimed to identify targets that are essential in both organisms but – when inhibited – target the parasite more effectively than the erythrocytes. Sleeping sickness is a deadly infectious disease for which new drugs are urgently needed. Current drugs have limited effectiveness, are highly toxic, and are experiencing rapidly increasing resistance^10^. Older drugs, such as suramin and melarsoprol have multiple targets, among which metabolic enzymes^11^. Eflornithine, a relatively recent drug, targets ornithine decarboxylase, an enzyme within the polyamine synthesis pathway^11^. *T. brucei* is a eukaryotic parasite that is transferred between mammals by bites of the tsetse fly. Before it eventually enters the central nervous system, the parasite proliferates extracellularly in the mammalian bloodstream. During this stage of its lifecycle, glycolysis is essential for survival as it is the only source of ATP. Only 50% inhibition of glycolysis is sufficient to kill trypanosomes^12^, which makes it a potent target pathway for antitrypanosomal drugs. Structural differences between human and trypanosome glycolytic enzymes make some of them attractive as drug targets^13^. Nevertheless, since glycolysis is also vital for the human host’s cells, drug selectivity remains a critical challenge. Notably, the erythrocytes that co-localize with *T. brucei* in the bloodstream also depend exclusively on glycolysis for their ATP and all their glycolytic enzymes are essential. Insufficient drug selectivity might therefore result in anemia^14^. These aspects make erythrocytes well-suited for testing the validity of the concept of network-based drug target identification.

We here analyzed two validated, data-driven kinetic models of glucose metabolism, one of the clinically relevant long-slender bloodstream stage of *T. brucei*^15^ and the other of human erythrocytes^16^. We quantified the network-based efficacy and selectivity of its enzymes as drug targets. Subsequently, we proved theoretically that the network-based component of drug selectivity is equally important as its structure-based component. Finally, we experimentally validated one of the most highly ranked drug targets: the plasma membrane glucose transporter. We showed in specially designed co-culture experiments of trypanosomes and erythrocytes that glucose-transport inhibitors do not need structural selectivity to target parasite metabolism and survival selectively. Finally, mammalian neuronal cells and hepatocytes were much less affected by such inhibitors than trypanosomes or malaria-infected erythrocytes.

## Results

### Ranking drug targets by kinetic modelling

**Figure 1:**
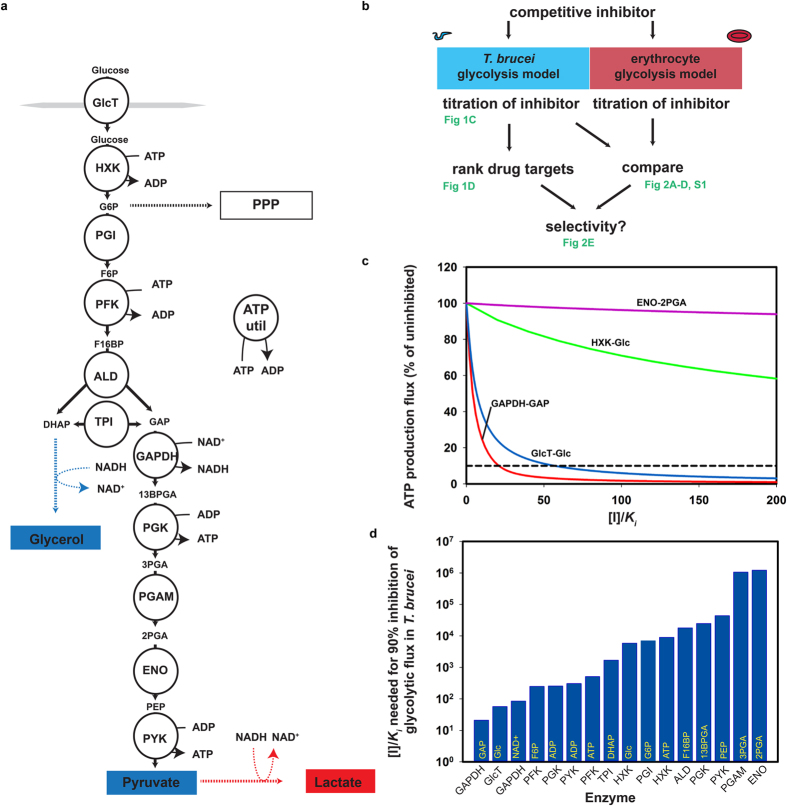
Simulating competitive inhibition of trypanosome glycolysis. (**a**) Outline of glycolysis and the reactions included in the in silico models. Circles show the enzymes. Blue parts are specific to bloodstream-form *T. brucei* and red parts are erythrocyte specific. The boxed and colored metabolites are secreted. Note that BSF *T. brucei* only produces substantial amounts of glycerol under anaerobic conditions^48^ and that in *T. brucei* the enzymes from HXK to PGK are sequestered in specialized peroxisomes, called glycosomes. ATP util: ATP utilization; GlcT: glucose transport; HXK: hexokinase; PGI: phosphoglucoisomerase; PFK: phosphofructokinase; ALD: aldolase; TPI: triose-phosphate isomerase; GAPDH: glyceraldehyde-3-phosphate dehydrogenase; PGK: phosphoglycerate kinase; PGAM: phosphoglycerate mutase; ENO: enolase; PYK: pyruvate kinase; PPP: pentose phosphate pathway (present in detail in both models); G6P: glucose 6-phosphate; F6P: fructose 6-phosphate; F16BP: fructose 1,6-bisphosphate; DHAP: dihydroxyacetone phosphate; GAP: glyceraldehyde 3-phosphate; 13BPGA: 1,3-bisphosphoglycerate; 3PGA: 3-phosphoglycerate; 2PGA: 2-phosphoglycerate; PEP: phosphoenolpyruvate. (**b**) Flowchart of the computational approach (**c**) The steady-state ATP production flux in the *T. brucei* model was simulated in the absence and presence of competitive inhibitors of a specific enzyme by increasing [I]/*K*_*i*_. The simulated inhibitor competed with the indicated substrate and the corresponding product. The dashed line indicates 90% flux inhibition. Glc: glucose. (**d**) For each glycolytic enzyme, inhibition of the ATP synthesis flux in *T. brucei* was simulated for an inhibitor competitive with the substrate indicated in yellow (and its product; see Table S1), and ranked on the basis of the amount of [I]/*K*_*i*_ needed to inhibit by 90%. The HXK inhibitor affected both the cytosolic and the glycosomal HXK fraction. Glc: glucose. See also Supplementary Fig. S1.

To identify potent, network-selective drug targets, we used two equally detailed computer models of glycolysis, one for *T. brucei*^15^ and one for the human erythrocyte^16^. The common glycolytic pathway with cell-type specific differences is schematically depicted in Fig. 1a. For each of the glycolytic enzymes in this pathway, we simulated the effect of competitive inhibitors (Fig. 1b). Both computer models consist of ordinary differential equations that describe how metabolite concentrations change over time. In these models the enzyme rates depend on the concentrations of substrates, products and effectors. The models contain mechanism-based enzyme-kinetic rate laws and have experimentally determined parameters. We used the models in the steady-state mode, when concentrations and fluxes have reached a time-independent, stable state. First, we investigated the effect of competitive inhibitors, since they represent the majority of existing mechanism- and structure-based inhibitors^17^.

A competitive inhibitor *I* affects an enzyme through its Michaelis-Menten constant (*K*_*m*_) for the competing substrate and product according to the formula


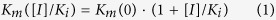


Here *K*_*i*_ is the inhibition constant of the inhibitor for the enzyme and *K*_*m*_(0) is the *K*_*m*_ in the absence of the inhibitor. An [*I*]/*K*_*i*_ of 1 effectively doubles the *K*_*m*_ for the specific competing substrate, which means a 33% reduction in enzyme activity at half-saturating substrate concentration. The lower the *K*_*i*_, the stronger the inhibitor affects the enzyme. The *K*_*i*_ depends on the structural affinity of the inhibitor for the active site of the enzyme. Varying the [*I*]/*K*_*i*_ ratio rather than the inhibitor concentration itself ensures that the simulations are independent of this structural affinity and hence independent of the actual inhibitor used.

**Figure 2:**
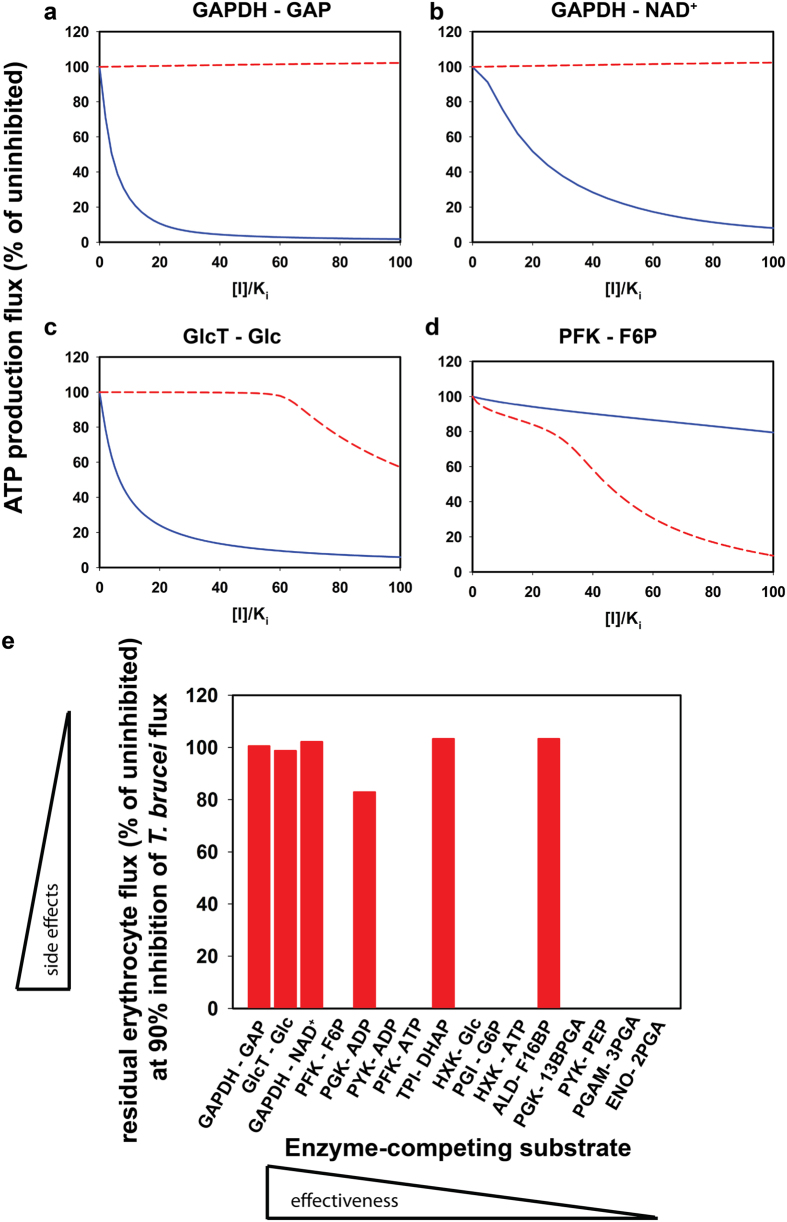
Simulating the differential inhibition of *T. brucei* and erythrocyte glycolysis. (**a**–**d**) Competitive inhibition was simulated in the *T. brucei* (blue, solid line) and erythrocyte glycolysis models (red, dashed line). The target enzyme and the competing substrate are indicated above each panel. Abbreviations as in Fig. 1. (**e**) At the [I]/*K*_*i*_ value that inhibited the flux of *T. brucei* by 90% (Fig. 1d), we calculated the residual glycolytic flux in the erythrocyte model. The higher the residual flux the fewer side effects can be expected. See also Supplementary Table S1, S2 and Figs S1 and S4.

Antitrypanosomal drugs that target glycolysis should in the first place exert a strong effect on the ATP production flux, which is exclusively glycolytic in the trypanosome. The effect of inhibitors on the *T. brucei* ATP production flux depended strongly on their target enzyme: e.g. an inhibitor of glyceraldehyde-3-phosphate dehydrogenase (GAPDH) or glucose transport reduced the ATP synthesis flux more than an inhibitor of hexokinase, which in turn was more effective than an enolase inhibitor (Fig. 1c). We used these titrations to calculate – for each combination of enzyme and competing substrate/product – which [*I*]/*K*_*i*_ was required to reduce the *T. brucei* ATP production flux by 90% (Fig. 1c) and sorted them from lowest to highest (Fig. 1d). The lower the [*I*]/*K*_*i*_ ratio needed for 90% inhibition of glycolytic flux, the lower the dose that will be required to kill the parasites and thus the more potent the target. The 90% cut-off is a safe choice, since already 50% inhibition is sufficient to start killing trypanosomes^12^. Qualitatively, the ranking was hardly affected by the precise cut-off, as can also be seen from the complete titration curves (Fig. 2 and Supplementary Fig. S1). For inhibitors of GAPDH – which compete with the enzyme’s substrate glyceraldehyde 3-phosphate (GAP) and with its product 1,3-bisphosphoglycerate (13BPGA) – a concentration of 35 times the *K*_*i*_ was sufficient to reduce the flux by 90%. In contrast, a competitive inhibitor of enolase required a dose of more than 10^6^ times its *K*_*i*_ to achieve the same effect. For some enzymes with multiple substrates the choice of the competing substrate affected the outcome: an inhibitor of pyruvate kinase (PYK) that competed with ADP and ATP achieved 90% flux inhibition at a markedly lower concentration than one that competed with phosphoenolpyruvate (PEP) and pyruvate (Fig. 1d). According to our predictions, the three best competitive inhibitors acted either on GAPDH (competitive with either GAP/13BPGA or NAD^+^/NADH) or on the glucose transporter in the plasma membrane (GlcT).

To avoid toxicity, putative drugs should have minimal effect on erythrocyte glycolysis. We therefore used the same competitive inhibitors for titrations in the erythrocyte model and the trypanosome model (Fig. 2a–d and Supplementary Fig. S1). Inhibition of the most potent targets in *T. brucei* – GAPDH and glucose transport – had only a limited effect on erythrocyte glycolysis (Fig. 2a–c), classifying them as highly selective network-based targets. The next most potent *T. brucei* inhibitor – acting on phosphofructokinase (PFK) and competitive with fructose 6-phosphate (Fig. 1d) – conferred unwanted network selectivity: a greater effect on erythrocyte glycolysis than on trypanosome glycolysis (Fig. 2d). For the [*I*]/*K*_*i*_ values that inhibited 90% of the *T. brucei* glycolytic flux (Fig. 1d) we calculated how the flux would be affected in erythrocytes (Fig. 2e). This confirmed that the three competitive inhibitors that were most effective in reducing *T. brucei* flux (the two GAPDH inhibitors and the GlcT inhibitor) did not affect erythrocyte flux. There were three other inhibitors that had moderate or negligible effects on erythrocyte glycolysis. However, for these inhibitors, 10- to 1000-fold higher doses (or lower *K*_*i*_) are needed to inhibit the trypanosome flux by 90% (cf. Fig. 1d), making them less suitable as potential drugs.

### Network‐based selectivity is just as important as structure and pharmacokinetics for the overall drug selectivity

We then studied the general principles underlying drug selectivity by adopting the framework of metabolic control analysis (MCA) (reviewed in ref. 18). The overall effect of a drug on cell function depends on: (i) the drug concentration to which the target enzyme is exposed, and which depends on pharmacokinetics; (ii) the structural affinity of the target enzyme for the drug; (iii) the local environment of the enzyme (e.g. competing metabolite concentrations) which affects the *in vivo* affinity of the target enzyme to the drug; and finally (iv) to which extent the target enzyme limits a vital metabolic process. MCA addresses perturbations in the mathematical limit to infinitely small parameter changes and provides therefore an approximation of real drug effects. The advantage is that we can apply it to integrate network-based and structure-based selectivitiy in general, mathematical terms. In MCA the effect of a small change in inhibitor concentration *d*[*I*]_*T*_ at the target site on a steady-state metabolic flux *J* is approximated by a response coefficient 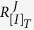. Here we use a partially normalized version of the response coefficient, to avoid division by zero at zero [*I*]_*T*_: 
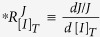
, which can be dissected into:


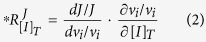


in which *v*_*i*_ is the rate of enzyme *i;* and where the partial derivative ∂ is taken by only changing the concentration of the inhibitor at constant values of metabolite concentrations. The total derivative indicated by the *d*’s refers to the effect of the altered enzyme rate *v*_*i*_ on the flux *J* through both direct and indirect effects.

Using the flux control coefficient:


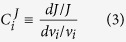


and the partially normalized elasticity coefficient:


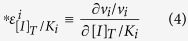


we rewrite equation 2 into:


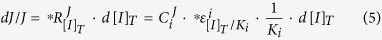


If we evaluate this equation at an infinitely small inhibitor concentration, *d*[*I*]_*T*_ can be replaced by [*I*]_*T*_. [*I*]_*T*_ relates to the inhibitor concentration in the serum [*I*]_*S*_ through a partition coefficient *P*_*I*_ = [*I*]_*T*_/[*I*]_*S*_, which depends on the uptake of the drug into the target cell. This leads to:


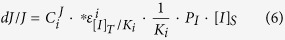


In Eq. 6 the flux control coefficient 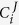 quantifies the extent to which a change in activity of the target enzyme *i* affects the steady-state flux *J*, i.e. the extent to which an enzyme limits the flux. The flux control coefficient involves adaptation of the entire pathway through changes in metabolite concentrations. The control coefficient is therefore a property of the entire metabolic network. The elasticity coefficient 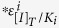 quantifies the direct sensitivity of the target enzyme towards a small addition of the inhibitor, normalized for the corresponding inhibition constant. Depending on the inhibition mechanism, the elasticity coefficient may depend on the metabolite concentrations prior to the addition of inhibitor. For instance, the effect of a competitive inhibitor is influenced by the concentration of the competing metabolite(s). In this sense, the elasticity coefficient is also part of the network response. The mathematical product of the flux control coefficient and the elasticity coefficient therefore quantifies the total network-based effect of the drug on the target cell. In contrast, the inhibition constant *K*_*i*_ quantifies the structure-based affinity of the target enzyme for the inhibitor. The *K*_*i*_ can be optimized by altering the chemical structure of the inhibitor and therefore embodies the structure-based drug effect. The partition coefficient *P*_*I*_ of the drug between serum and target site measures the pharmacokinetics. Although *P*_*I*_ can be optimized through structure-based drug design, it also depends on the ADME (Administration-Distribution-Metabolism-Excretion) network.

Eq. 6 is valid for both the pathogen and the host. Therefore, the drug selectivity can be defined as follows:


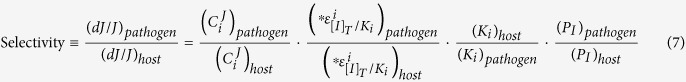


The serum concentration [*I*]_*S*_ drops out of this equation since it is the same for parasite and host. From Eq. 7 it follows immediately that network-based determinants of selectivity (ratios of control and elasticity coefficients between pathogen and host) are equally important for overall drug selectivity as structure-based selectivity (the ratios of inhibition constants) and pharmacokinetics (the ratio of partition coefficients).

Zooming in on the network-based selectivity, we calculated the flux control coefficients and elasticity coefficients for putative target enzymes in the glycolysis models of *T. brucei* and human erythrocytes. The parasite-host ratios represent the network-based part of the selectivity (Supplementary Table S2). Indeed, the most potent network-based targets identified above (glucose transport and GAPDH) rank favorably. According to this MCA-based analysis at low drug concentrations, the glucose transporter ranked even better than GAPDH (Supplementary Table S2; final column), showing that the conditions may affect the precise ranking. Given that conditions *in vivo* may be variable, we do not think that this subtle difference should favour one target over the other. The underlying ratios of flux control and elasticity coefficients (Table S2) enable dissection of the network-based selectivity. In the case of the glucose transporter, the network-based selectivity can be attributed to the high ratio of flux control coefficients of *T. brucei* and human erythrocytes (5.8 ∙ 10^3^), while the ratio of elasticity coefficients was close to 1 and hence conferred no selectivity. Indeed the flux control coefficient in trypanosomes has previously been shown to be high^19^, while the erythrocyte transporter seems to act close to equilibrium and therefore does not limit the flux^20^,^21^. For GAPDH, the flux control coefficients also dominated the network-based selectivity. The elasticity coefficients even conferred some selective damage to erythrocytes if the inhibitor were to compete with NAD^+^ and NADH. Analogously, all inhibitors of ATP- or ADP-utilizing enzymes suffered from an unfavorable elasticity ratio if they were to compete with these cofactors. In general, the elasticity coefficients increase if the competing metabolite concentrations are reduced. When considering the possible design of drugs that compete with NADH or ATP, their potency might be improved substantially by combining them with a drug that inhibits the biosynthesis of these coenzymes.

### From in silico to *in vitro*: co-cultures of *T. brucei* with erythrocytes

**Figure 3:**
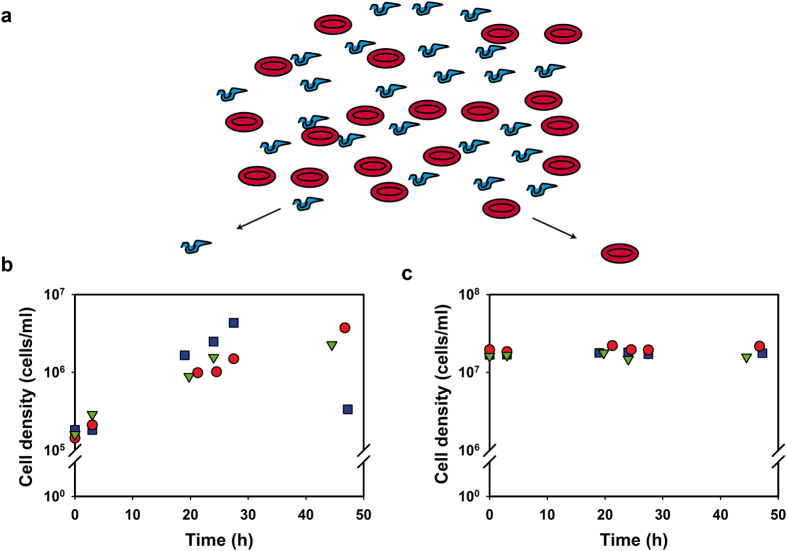
Co-cultures of *T. brucei* and erythrocytes. (**a**) Schematic representation of the co-cultures of *T. brucei* (in blue) and erythrocytes (in red) in HMI-9 medium. The cell density of trypanosomes (**b**) and erythrocytes (**c**) are shown for three independent co-cultures (each given a different color and symbol). These are the control cultures for the experiments shown in Fig. 4. See also Supplementary Fig. S2.

To test the model predictions directly, we developed novel co-culture experiments of *T. brucei* and human erythrocytes (schematically depicted in Fig. 3a). We used the *T. brucei* culture medium (HMI-9) in which erythrocytes also survived (Supplementary Fig. S2). Fresh erythrocytes were washed and resuspended in HMI-9, together with an aliquot from an exponentially growing trypanosome culture. As in monocultures^22^, *T. brucei* cell numbers increased exponentially for 24 h (Fig. 3b). Growth then slowed down until a maximum cell density of 3–4 × 10^6 ^cells/ml was reached; after longer cultivation, trypanosome densities started to decrease (Fig. 3b). In the same co-cultures, erythrocyte cell densities stayed constant throughout the experiment (Fig. 3c). We note that the trypanosome to erythrocyte ratio in our co-cultures (1 in 10–100) is much higher than in patient blood^23^. This is necessary to get reliable cell counts and analyse parasite metabolism (below).

Pyruvate is secreted as the major end-product of *T. brucei* glycolysis^22^, while erythrocytes metabolize glucose to lactate (Fig. 1a). Since neither cell-type has mitochondrial ATP production, these fluxes can be used to quantify their ATP production fluxes. To test whether we could use pyruvate production as a specific measure of *T. brucei* glycolysis and lactate production as a specific measure of erythrocyte glycolysis, we measured pyruvate production in erythrocyte monocultures and found it to be negligible (Supplementary Fig. S2). The lactate production flux in erythrocyte monocultures was two orders of magnitude lower than the pyruvate production flux of *T. brucei* (see controls in Supplementary Fig. S3 and ref. 22). This low lactate production (~1 nmol/min/10^8^ cells) was in agreement with measurements in erythrocytes by others^24^. Furthermore, addition of 5 mM pyruvate increased the lactate production flux of erythrocytes only marginally (Supplementary Fig. S2), suggesting that the pyruvate produced by the trypanosomes should not affect lactate production by the erythrocytes. We did not detect lactate production in our trypanosome monocultures, which is consistent with the absence of a lactate dehydrogenase in the *T. brucei* genome. Finally, high lactate concentrations of up to 6.2 mM did not affect *T. brucei* growth (Supplementary Fig. S2).

### Glucose-transport inhibition affects *T. brucei* metabolism selectively in co-cultures

**Figure 4:**
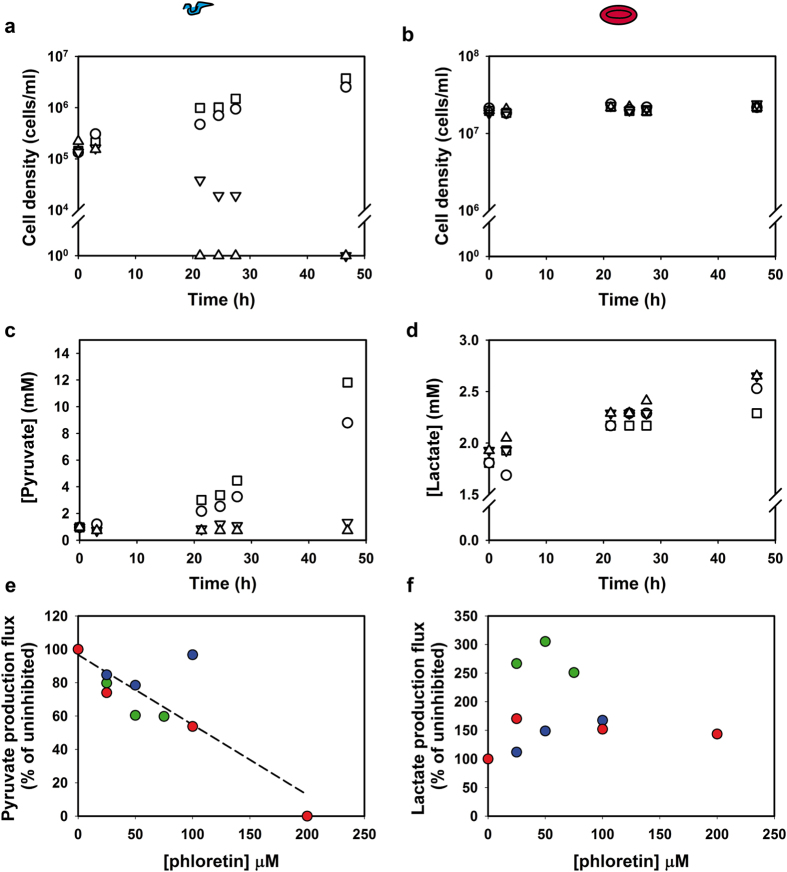
Effects of glucose-transport inhibition on co-cultures of *T. brucei* and erythrocytes. Co-cultures were treated with various concentrations of phloretin in 0.14% ethanol or with 0.14% ethanol only (control): □ ethanol control, ◯ 25 μM, ▽ 100 μM, △ 200 μM phloretin. Ethanol was shown not affect metabolism in the co-cultures (see Fig. S2 and ref. 12). (**a**) Cell density of trypanosomes in the co-cultures. The culture treated with 100 μM phloretin had no live trypanosomes at 47 h, and for 200 μM phloretin trypanosomes were dead at the 21 h time point. (**b**) Cell density of erythrocytes in the co-cultures. (**c**) Concentration of pyruvate in the co-cultures. (**d**) Concentration of lactate (the metabolic end-product of erythrocytes) in the co-cultures. (**e**) Pyruvate fluxes were calculated over the first 20–24 h (the exponential growth phase for trypanosomes in the control cultures). Each color denotes an independent experiment. The red data points are from the same experiment as shown in panel c and d. Fluxes are displayed relative to the flux in the corresponding control co-culture. The dashed line represents a linear fit through all data points (R^2^ = 0.73). A linear fit without the 200 μM point and the outlier at 100 μM is given in Supplementary Fig. S3 and has an R^2^ = 0.86 (**f**) Lactate production fluxes were calculated over 50 h. Each color denotes an independent experiment and the color coding corresponds to that of Fig. 4e. Fluxes are relative to the flux in the corresponding control co-culture. See also Supplementary Figs S2 and S3.

According to our in silico analysis, the best-ranking drug targets were GAPDH and the glucose transporter. Although inhibitors of *T. brucei* GAPDH exist^13^, for a stringent test of the network-based selectivity we decided to target glucose transport. This target has the advantage drug uptake is not required. We set out to test the model’s prediction that inhibition of glucose transport will affect *T. brucei* glycolysis without harming that of the erythrocytes, even if the inhibitor is not selective for the trypanosome transporter THT1 over the erythrocyte transporter GLUT1^25^. Erythrocytes also express the fructose transporter GLUT5^26^, but there is no fructose in the medium. We incubated co-cultures with 0–200 μM of phloretin, a competitive inhibitor of glucose transport in both trypanosomes and erythrocytes^12^,^19^. Figures 4a–d show growth and metabolite production in one representative experiment out of three, and Fig. 4e and f summarize the fluxes as determined in all three experiments. Phloretin reduced the growth of *T. brucei* only slightly at 25 μM, but at 100 μM the trypanosome numbers decreased after 20 h (Fig. 4a). When subjected to 200 μM phloretin, no living trypanosomes were found after the 3 h time point. In contrast, none of these phloretin concentrations affected the erythrocyte density in the co-cultures (Fig. 4b). The trypanosomal pyruvate production was also affected strongly by phloretin treatment (Fig. 4c and e), with hardly any pyruvate production at and above 100 μM phloretin. Phloretin did not inhibit the lactate production by erythrocytes (Fig. 4d and f), suggesting that the treatment does not compromise their metabolic health. The lactate flux was at the limit of what we could quantify. We could not, however, boost the lactate production by using higher erythrocyte cell densities: despite elaborate washing of erythrocytes, trypanosomes were unable to grow at erythrocyte densities above 2 × 10^7 ^cells/ml (Supplementary Fig. S2). This might be due to the presence of residual amounts of the trypanosome lytic factor in the erythrocyte preparations from human blood^27^. We note that the uninhibited lactate flux was lower in co-cultures than in erythrocyte monocultures. The addition of phloretin – even at the low concentration of 25 μM, where it hardly affected pyruvate production by trypanosomes – increased lactate production in co-cultures up to the level observed in erythrocyte monocultures in the absence of phloretin. This suggests that trypanosomes had a negative effect on erythrocyte metabolism, which was rectified by inhibition of trypanosome glycolysis. This is not due to the pyruvate produced by trypanosomes, since addition of pyruvate did not affect lactate production in erythrocyte monocultures (above).

### Glucose-transport inhibition selectively affects *T. brucei* survival in co-cultures

**Figure 5:**
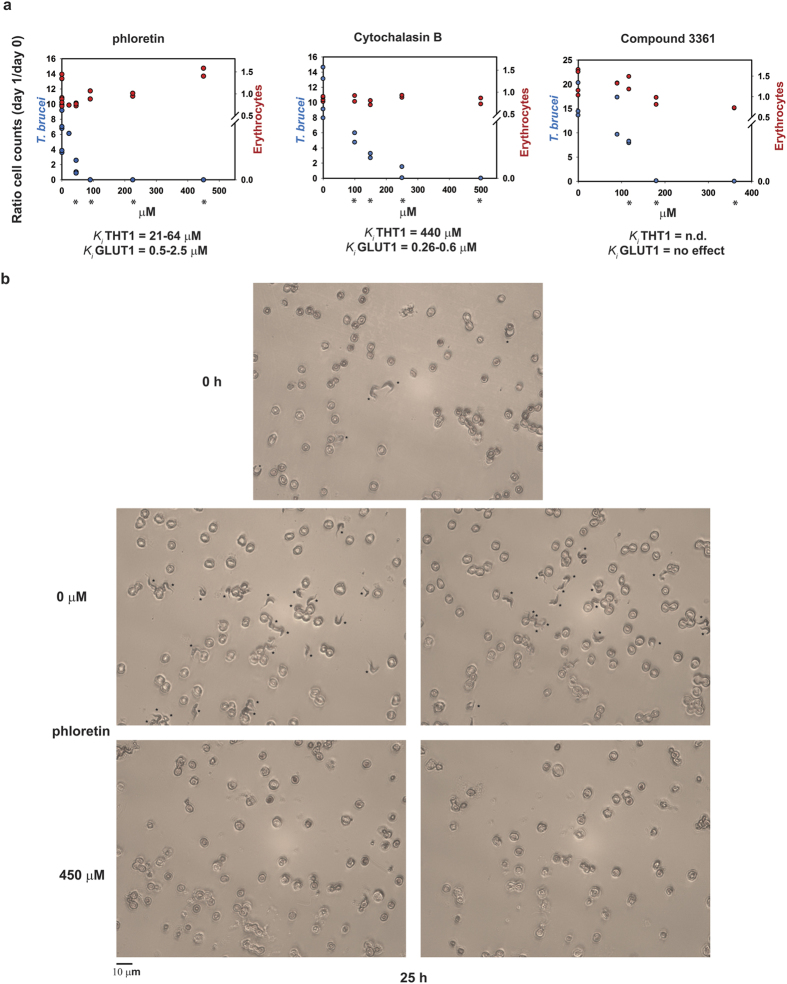
Differential survival in co-cultures treated with three different glucose-transport inhibitors. (**a**) Cells in the co-cultures were counted at the start of the experiment (day 0) and on day 1 (after 25–28 hours), and the ratio was plotted. Published inhibition constants (*K*_*i*_ values) are indicated below the graph for the trypanosome glucose transporter (THT1)^19^,^49^ and the erythrocyte transport (GLUT1)^50^,^51^,^52^. N.d. = not previously determined. The left axis shows the ratio for the trypanosomes in the co-culture; the right axis shows the ratio for the human erythrocytes in the co-cultures. Each concentration of inhibitor was tested at least twice on independent cultures. Each graph shows the combined results of at least two different experiments, resulting in ≥4 independent controls. For phloretin: Cell densities at the start were 2–7 × 10^5^/ml for *T. brucei*, and ~5 × 10^6 ^cells/ml for erythrocytes. All co‐cultures were treated with ≤0.1% v/v ethanol (solvent). For cytochalasin B: Cell densities at the start were 1–4 × 10^5^/ml for *T. brucei*, and ~1 × 10^7 ^cells/ml for erythrocytes. Co‐cultures were treated with ≤0.7% v/v ethanol (solvent). Only the co‐culture that was treated with 500 μM cytochalasin B had a concentration of 1.4% ethanol due to the maximum solubility of cytochalasin B. This ethanol concentration had a minimal effect on the *T. brucei* growth rate. For compound 3361: Cell densities at the start were 1.5 × 10^5^/ml for *T. brucei*, and 0.5–1 × 10^6 ^cells/ml for erythrocytes. Co‐cultures were treated with ≤0.2% v/v dimethyl sulfoxide (solvent). Asterisks below the graphs indicate for trypanosomes at which concentrations the mean of the data-points differs from the mean of the controls with a P-value < 0.05 in a two-tailed t-test assuming unequal variances. (**b**) Microscope images of Giemsa-stained smears of a co-culture before treatment with phloretin (t = 0) and two co-cultures treated with solvent (EtOH) or 450 μM of phloretin for 25 hours. Asterisks mark the trypanosomes in the cultures. The experiments in this figure were performed independently of the metabolic experiments shown in Fig. 4. Supplementary Fig. S3 shows that at these inhibitor concentrations the lactate production flux by erythrocytes is unaltered compared to that of uninhibited erythrocytes. See also Supplementary Fig S3.

Subsequently, we monitored cell growth and survival over a broader phloretin concentration range and for two additional glucose-transport inhibitors: cytochalasin B and compound 3361 (Fig. 5a). Cytochalasin B is an inhibitor of erythrocyte and trypanosome glucose transport (ref. 28 and Supplementary Fig. S3). Compound 3361 is a glucose analog that was originally designed against the malaria parasite *Plasmodium falciparum*^29^, where it was selected because it did not affect the erythrocyte transporter GLUT1. Each of these inhibitors reduced trypanosome proliferation in a dose-dependent manner without affecting erythrocyte density. All trypanosomes were killed in co-cultures that were treated with ≥100 μM phloretin, ≥250 μM cytochalasin B or ≥190 μM compound 3361 (Fig. 5a). Figure 5b shows microscopy images of control (solvent-treated) co-cultures and co-cultures treated with 450 μM phloretin for 25 h. The latter were entirely cleared of trypanosomes. Erythrocytes appeared unaffected, although we cannot draw any conclusions on erythrocyte health from these pictures. In separate experiments we showed that at this phloretin concentration, lactate production of erythrocytes in unaffected (Supplementary Fig. S3). As a positive control to show that metabolic inhibitors can affect erythrocyte survival we treated erythrocytes with 2-phosphoglycolate, an inhibitor of erythrocyte pyruvate kinase^30^ (Supplementary Fig. S3). The highest concentration (24 mM) killed all erythrocytes within one day, in agreement with the known detrimental effect of insufficient pyruvate kinase levels in erythrocytes^14^.

For phloretin and cytochalasin B, we confirmed that concentrations that were lethal to trypanosomes (Fig. 5a), did not affect lactate production flux in erythrocyte monocultures (Supplementary Fig. S3). Since phloretin and cytochalasin B have been reported to be more active against the erythrocyte glucose transporter GLUT1 than against the *T. brucei* counterpart THT1 (Fig. 5a and ref. 28), our results are a prominent example of how a strong network-based selectivity can even overrule unfavorable selectivity at the molecular level (cf. Eq. 7).

### The broader perspective: phloretin and cytochalasin B only moderately affect neurons and hepatocytes, but strongly affect *Plasmodium falciparum*

We did a pilot study to test the systemic effect of phloretin on *T. brucei* in mice infected with *T. brucei*. However, despite initial clearing of the infection (Supplementary Fig. S5), validating our modelling results, phloretin had no lasting effect due to its poor pharmacokinetics (ref. 31 and Supplementary Table S3).

**Figure 6:**
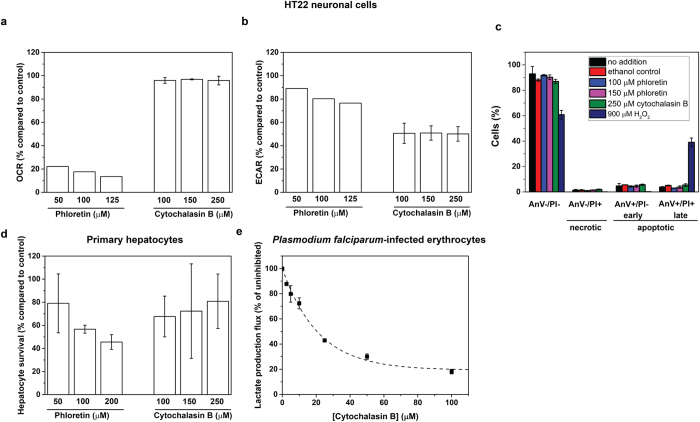
Effects of phloretin and cytochalasin B on neuronal cells, hepatocytes and malaria-infected erythrocytes. (**a**) Oxygen consumption rate (OCR) in HT22 neuronal cells after treatment with phloretin ((n = 1, but based on 6 technical replicates of which the standard deviation was 0.9–1.6%) and cytochalasin B (n = 2; two biological replicates with 3 technical replicates each – shown is the mean with standard deviation compared to vehicle-control compared to vehicle-control). Raw data before and after inhibition is shown in Supplementary Fig. S7 (**b**) Extracellular acidification rate (ECAR) in neuronal HT22 cells after oligomycin treatment following treatment with phloretin (n = 1, but based on 6 technical replicates of which the standard deviation was 2.5–5%)) or cytochalasin B (n = 2; two biological replicates with 3 technical replicates each – shown is the mean with standard deviation compared to vehicle-control). (**c**) Cell death assay on neuronal HT22 cells after 5 hours of indicated treatment. Healthy cells exclude propidium iodide (PI) and only apoptotic cells are positive for AnnexinV (AnV+). Shown is the mean and standard deviation of three incubations. 900 μM H_2_O_2_ is known to evoke apoptosis and is a positive control (**d**) Survival based on cell impedance measurements of primary rat hepatocytes after 20 hours of treatment with phloretin or Cytochalasin B compared to vehicle control. Shown is the mean of two biological replicates with standard deviation. (**e**) Lactate production flux in erythrocytes infected with *Plasmodium falciparum* enriched for parasites in the trophozoite stage. The lactate produced was followed over 1 hour (5 time points) in the absence or presence of cytochalasin B. The uninhibited flux equals 0.069 fmol/min/erythrocyte. Each datapoint is based on at least two biological replicates and three technical replicates; error bars show standard error of the mean. The dashed line is a fitted exponential decay function. See also Supplementary Figs S6 and S7.

Two host cell types that are expected to be sensitive to glucose-transport inhibition are neuronal cells, the brain being the major consumer of glucose in the body, and hepatocytes, since the liver plays a crucial role in whole-body glucose homeostasis. We therefore tested the effect of the inhibitors on neuronal cell line HT-22 and primary rat hepatocytes. Phloretin, but not cytochalasin B, had a strong effect on the oxygen consumption rate (OCR) of HT-22 cells (Fig. 6a). Indeed, inhibition of mitochondrial respiration is a known side effect of phloretin^32^, making this inhibitor less suitable for the testing of glucose transport inhibition in neurons and hepatocytes. This side effect was irrelevant in erythrocytes, which do not contain mitochondria. Subsequently, the effect on glycolytic flux was measured as extracellular acidification rate (ECAR) in the presence of the oxidative phosphorylation inhibitor oligomycin. 125 μM of phloretin, a dosage sufficient to kill trypanosomes (cf. Fig. 5a), inhibited the glycolytic flux in HT-22 cells by 25% and 250 μM cytochalasin B by 50% (Fig. 6b). At similar concentrations phloretin and cytochalasin B did not kill HT-22 cells at all (Fig. 6c), showing clear selectivity of the drugs against trypanosomes versus HT-22 cells. Hydrogen peroxide was added as a positive control and clearly triggered apoptosis in HT-22 cells (Fig. 6c).

To investigate how glucose transport inhibition affects liver cells, we first simulated glycolysis with a comprehensive kinetic model of human hepatocytes, which includes hormonal effects and glycogen content. Earlier analyses of this model had shown that the hepatic glucose transporter GLUT2 exerted no flux control over a range of glucose concentrations^33^, suggesting that hepatocytes are not vulnerable to glucose transport inhibition. Using the same model, we calculated that hepatocyte glucose production and glycolytic flux where hardly affected in the range of inhibitor concentrations that kill trypanosomes (Supplementary Fig. S6). As an *in vitro* test we monitored the change in cell impedance of primary hepatocyte cultures over 20 hours in the absence or presence of glucose transport inhibitors (Fig. 6d). Impedance is caused by adherence of cells to the bottom and used as a proxy for cell proliferation and shape. 60% impedance was maintained during treatment with 100 μM of phloretin (a concentration that kills trypanosomes within a day (Fig. 5a)), and 70% in the presence of cytochalasin B. These results show that, although hepatocyte viability is clearly somewhat affected by these inhibitors, glucose transport inhibition has network-selectivity against trypanosomes also compared to hepatocytes.

Finally, we tested the effect of glucose transport inhibition on the malaria parasite *Plasmodium falciparum*. In contrast to *T. brucei, P. falciparum* is an intracellular parasite that invades erythrocytes. Recently, computational modelling suggested that also in *Plasmodium* trophozoites (the blood stage) the glucose transporter is the preferred network-based target and this was confirmed in isolated trophozoites^34^. In contrast to *T. brucei*, the malaria parasite produces lactate. This lactate production is up to 100-fold higher than that of its host cell^35^. When *Plasmodium*-infected erythrocytes were subjected to cytochalasin B, a strong and dose-dependent reduction of the lactate production flux was found (Fig. 6e). Since the lactate production flux of infected erythrocytes is dominated by the contribution of the intracellular parasites, this indicates that glucose transport inhibition is also prominent target in *Plasmodium* when residing in its host cell.

## Discussion

In this paper we have shown – both theoretically and experimentally – how it is possible to kill a pathogen selectively, with minimal effect on the host, by targeting a metabolic pathway that is essential for host and pathogen alike. Although targeting a unique pathogen protein may open powerful avenues for selective inhibition, uniqueness itself does not make it a potent drug target against the pathogen. A drug against a target that is not essential for survival of the pathogen will not kill it. Furthermore, if near-complete inhibition of the unique target would be needed in order to kill the pathogen, the required inhibitor dosage would increase the risk of off-target side-effects to the host. Essentiality of the target pathway and relatively high flux control of the specific target in the parasite thus need to be the first criteria for potential targets. Identification of a pathogen-specific target that meets these criteria enables design of drugs with strong molecular selectivity.

Our study shows that very promising targets can exist outside the realm of the unique proteins. Glycolysis is an essential pathway for bloodstream-form *Trypanosoma brucei* and a moderate inhibition (50%) is sufficient to kill the parasite^12^. This makes it a promising target pathway for antitrypanosomal drugs. However, *T. brucei* glycolysis does not contain unique parasitic proteins (although some are evolutionary and structurally distant from their human counterparts (see ref. 13 and references therein). Moreover, glycolysis is also essential for many human cell types. We have demonstrated that this need not be an obstacle: quantitative differences between trypanosome and erythrocyte lead to strong network-based selectivity. This network-based selectivity depends equally on the flux control exerted by the target enzyme and on the *in vivo* elasticity (sensitivity) of the target enzyme towards the drug. The latter depends on the degree of saturation of the enzyme with its substrates and products. The generality of this principle makes it applicable to any disease in which specific cell types must be targeted selectively. We have further shown that this network-based selectivity is just as important as selectivity based on the structure of the molecular target (Eq. 7).

Here we validated model simulations for the network-selectivity of glucose transport inhibition towards *T. brucei* over erythrocytes. We also showed that inhibition of pyruvate kinase is indeed detrimental to erythrocytes, as predicted by the corresponding model. With respect to some other targets, both kinetic models had previously been validated extensively. For the *T. brucei* model, apart from the glucose transporter itself^19^, the effect of reduction of various other enzyme activities had been validated by RNAi knockdowns^9^. The erythrocyte model has been validated by comparing predictions to clinical data of inborn enzyme deficiencies^36^.

Drug design that is mechanistic and structure-based will often lead to the development of competitive inhibitors. Several drugs in clinical use are competitive enzyme inhibitors, such as the phosphodiesterase inhibitor Viagra and the bcl-abl kinase inhibitor Gleevec^17^. This was why we focused on competitive inhibitors first. The values observed for the elasticity coefficients suggest, however, that other modes of drug action may lead to a different network-based selectivity. For instance, in the computer models a competitive inhibitor of aldolase was calculated to be somewhat selective against erythrocytes (Supplementary Fig. S1) due to a lower concentration of the competing substrate fructose-1,6-bisphosphate (0.0010 mM in erythrocytes versus 13 mM in trypanosomes). This caused a higher elasticity in the erythrocyte (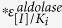 was −6.7 × 10^−2^ in erythrocytes versus −3.0·10^−3^ in trypanosomes), conferring a 22-fold selectivity towards the erythrocyte enzyme. Since uncompetitive inhibitors become more effective at higher substrate concentrations^7^, uncompetitive inhibitors of aldolase should be selective against trypanosomes, which we confirmed computationally (Supplementary Fig. S4).

Each host cell type has its own characteristic metabolic network. Indeed, our *in vitro* tests on primary neurons and liver cells show that these cell types are more affected by glucose transport inhibition than erythrocytes, but still markedly less than trypanosomes. With respect to toxicity for these cells it may therefore be useful to enhance the network selectivity with molecular selectivity. Molecular side-effects against the hepatocyte and brain transporters GLUT2 and GLUT3, respectively, should then be more important to consider than those against the erythrocyte transporter GLUT1. In addition, a chronic and severe deficiency of GLUT1 is known to affect the brain, since this transporter is also essential for glucose uptake through the blood-brain barrier^37^, suggesting that a combined network- and structure-based approach will also for GLUT1 be the most effective. Realistically, however, a more comprehensive analysis of different host cell types will be needed. Although such an endeavor may be challenging, it is warranted by the fact that glycolysis is a promising target not only in trypanosomes, but also in various parasites including *Plasmodium*, as well as in certain tumors. Along these lines, we showed that glucose transport inhibition strongly affected metabolism in *Plasmodium*-infected erythrocytes. A characterization of host-cell network sensitivity can be reused for all these applications. In this light our results should be viewed as proof of principle that network selectivity is possible. Where necessary and possible, selectivity may be increased by exploiting structural differences between pathogen and host targets, eliciting synergy between network- and structure-based selectivity (cf. Eq. 7). The results obtained with compound 3361, which was selected for absence of an effect against the erythrocyte glucose transporter GLUT1^29^, suggest that the latter is feasible. This development may well be boosted by the elucidation of the crystal structure of GLUT1^38^.

A strong advantage of the selection of drug targets from a pathway operating in both pathogen and host is that it allows compound re-purposing. For instance, anticancer compounds that were discarded because of their limited effect on tumors, can now be re-tested for an effect on the high-ranking enzymes within trypanosome glycolysis (Fig. 1d).

The same approach will be crucial for identifying selective drug targets for the treatment of certain types of cancer. Since cancer cells originate from their host, their genome only encodes pathways that also operate in healthy cells. Upregulated and altered metabolism has been re-recognized as a hallmark of cancer and is back in focus as a target for anticancer drugs^39^. Differences in gene expression levels between tumor cells and healthy cells may be exploited to optimize network-based selectivity. We recommend that the strategy described in this paper be implemented in the development of potent drugs that cause minimal collateral damage to host tissues.

## Methods

### Computer models and code availability

For *T. brucei* glycolysis, we used the model version C from Kerkhoven *et al*.^15^ (availabe as a supplement to this reference), i.e. the model that contains the glycosomal ribokinase, but without the fructose branch. For the erythrocyte model by Holzhütter^16^ we used the curated SMBL file from BioModels^40^. The steady-state fluxes of this model were the same as reported in Table 1 of ref. 16.

Simulations were carried out in the program COPASI (version 4.11; build 65)^41^. The rate through the ATP utilization (ATPase) reaction was monitored as the output flux. At steady state this rate equals the net ATP production flux.

### Inhibitors

Phloretin was purchased from Sigma and Cytochalasin B from Serva. They were dissolved in 70% ethanol. Compound 3361 (3-*O*-[undecyl-10-en]-1-yl-D-glucose)^29^ was a kind gift from Dr. H. Staines and Dr. S. Krishna and was dissolved in DMSO. Control cultures were treated with the same amount of solvent as the corresponding inhibitor-treated cultures. The final solvent concentration in the cultures was ≤0.35% (v/v) ethanol for phloretin experiments, ≤0.7% ethanol (v/v) for cytochalasin B experiments and ≤0.2% (v/v) DMSO for experiments with compound 3361, unless indicated otherwise.

### Human blood

5 mL blood was drawn from a single subject by venipuncture into a Becton Dickinson Vacutainer containing heparin. A 20–200 μl aliquot of this, containing predominantly erythrocytes, was washed twice in more than 10 volumes of sterile isotonic buffer (25 mM HEPES, 1 mM NaH_2_PO_4_, 115 mM NaCl, 10 mM KCl, 2 mM MgCl_2_, pH = 7.5; ref. 24) and taken up in HMI-9, the culture medium for trypanosomes. Erythrocyte densities were determined manually by counting in a Bürker hemocytometer. We submitted our study to the medical ethics committee of the VUmc who found no ethical issues attached to this research.

### *T. brucei* cultivation

Monomorphic bloodstream-form *T. brucei* strain 427 (cell line 449)^42^ (obtained from Prof. P. Michels, then at Université Catholique de Louvain, Brussels) was cultured in HMI-9 supplemented with 10% fetal calf serum (Invitrogen) and 0.2 μg/ml phleomycin (Cayla) in a water-saturated incubator with 5% CO_2_ at 37 °C as described previously^42^. Cell densities were determined manually by counting in a Bürker hemocytometer. Only motile trypanosomes were counted. Cultures were maintained in exponential growth through dilution (i.e. between 1 × 10^5^ and 3 × 10^6 ^cells/ml). The cell line was not authenticated and cells have not been tested for mycoplasma contamination.

### Co-cultures of human blood and *T. brucei*

The erythrocytes were resuspended in HMI-9 medium, inoculated with *T. brucei* in HMI-9 medium and incubated as above. During the experiments glucose was not depleted.

### Metabolite measurement

Metabolite samples were taken from the culture, instantly quenched by addition of 1/10 volume of ice-cold 35% (v/v) perchloric acid (PCA). PCA-treated samples were snap-frozen in N_2_ (l) and stored at −80 °C. After thawing, the samples were neutralized by addition of 1/10 volume of an ice-cold solution of 5 M KOH in 0.2 M MOPS. After 10 minutes incubation on ice, the precipitated proteins were removed by centrifugation. Enzymatic assays for pyruvate^43^ and lactate^44^ were performed on the supernatant using a VITALAB Selectra E chemistry analyzer (INstruchemie, Delfzijl, The Netherlands). Time points were chosen such that the increase of both pyruvate and lactate in time could be quantified.

### Flux calculations

Pyruvate production fluxes were calculated over the first 20–24 h as described previously^12^ at phloretin concentrations up to 50 μM, the flux was calculated by multiplying the specific growth rate with the slope of the plot of pyruvate concentration versus cell density. At higher concentrations, when growth was severely affected, the slope of the pyruvate concentration against time was divided by the average trypanosome cell density. Lactate production fluxes were calculated over 50 h by dividing the slope of the lactate concentration versus time by the erythrocyte cell density (which remained constant).

### Microscopy

Smears of co-cultures were made on glass slides. Cells were fixed for 30 seconds in methanol and subsequently stained for 25 minutes in a 5% Giemsa solution in a phosphate-based buffer (6 mM KH_2_PO_4_, 4.3 mM Na_2_HPO_4_·H_2_O). Slides were washed three times with tap water and dried in air. Images were acquired using a Leica DM-LB microscope equipped with Leica Application Suite 3.8.0 build 818.

### Cell Culture of neuronal HT-22 Cells

HT-22 cells, which were derived from immortalized hippocampal neurons and obtained from Dr. Schubert, Salk Institute, San Diego, were cultured in Dulbecco’s modified Eagle’s medium (Invitrogen, Karlsruhe, Germany) supplemented with 10% heat-inactivated fetal calf serum, 100 U/ml penicillin, 100 μg/ml streptomycin, and 2 mM glutamine. The cell line was not authenticated but was tested to be mycoplasma-free.

### Measurements of Oxygen Consumption Rate (OCR) and Extracellular acidification rate (ECAR)

For OCR and ECAR measurements we used an XF96 Extracellular Flux Analyzer (Seahorse Bioscience, North Billerica, MA), which directly records the OCR in cells that remain attached to the culture plate by using calibrated optical sensors. The OCR/ECAR recordings were carried out as previously described with minor modifications^45^.

### Cell death analysis

Cells were seeded in 24-well plates (60,000 cells/well) for 24 h and challenged with different concentrations of phloretin and cytochalasin B. Cell viability was quantified after 5 h by AVPI staining and subsequent FACS-analysis. Cells were harvested by using Trypsin/EDTA, washed once in PBS and stained according to the manufacturer’s protocol (Annexin-V-FITC Detection Kit, PromoKine, Promocell, Heidelberg, Germany). Afterwards stained cells were analyzed by FACS-analysis. Annexin-V-FITC was excited at 488 nm and emission was detected through a 530 ± 40 nm band pass filter. Propidium iodide was excited at 488 nm and fluorescence emission was detected using a 680 ± 30 nm band pass filter. At least 10,000 gated events per sample were analyzed.

### Isolation of primary rat hepatocytes

Hepatocytes were isolated from male Wistar rats (220–250 g) by a two-step collagenase perfusion procedure as described previously^46^. Experiments were performed following the guidelines of the local Committee for Care and Use of Laboratory Animals of the University of Groningen. Cell viability was determined by trypan blue staining and exceeded 85%.

### Real-time monitoring of primary hepatocyte viability

10^4^ freshly isolated primary hepatocytes were plated per well of 0.20 cm^2^ on xCELLigence E plates with interdigitated gold microelectrodes to constantly record cell confluency by impedance measurement, according to manufacturer’s instructions^47^. The primary hepatocytes were grown in William’s E medium (Life Technologies Ltd; Breda, The Netherlands) supplemented with 50 μg/mL gentamycin (Life Technologies Ltd) and penicillin-streptomycin-fungizone (Lonza, Verviers, Belgium). During the attachment period (4 h) 50 nmol/L dexamethasone (Sigma, St Louis, USA) and 5% fetal calf serum (Life Technologies Ltd) were added to the medium. Cells were cultured in a humidified incubator at 37 °C and 5% CO_2_. Treatment was started 4 h after attachment. Results were recorded and analyzed by RTCA Software. Cell index at t = 20 h was compared to that at t = 0 h (addition of compound) and normalized to the ethanol-control (0.7% v/v).

## Additional Information

**How to cite this article**: Haanstra, J. R. *et al*. Targeting pathogen metabolism without collateral damage to the host. *Sci. Rep.* **7**, 40406; doi: 10.1038/srep40406 (2017).

**Publisher's note:** Springer Nature remains neutral with regard to jurisdictional claims in published maps and institutional affiliations.

## Supplementary Material

Supplementary Information
