# Supplementary material for: Targeting pathogen metabolism without collateral damage to the host
Source: Sci Rep. 2017 Jan 13;7:40406. doi: 10.1038/srep40406 (PMC5234033; doi:10.1038/srep40406)
Supplement: Supplementary Information [file srep40406-s1.pdf]

Supplementary information to

Targeting pathogen metabolism without collateral damage to the host

Jurgen R. Haanstra, Albert Gerding, Amalia M. Dolga, Freek J. H. Sorgdrager, Manon Buist-Homan, François du Toit, Klaas Nico Faber, Hermann-Georg Holzhütter, Balázs Szöör, Keith Matthews, Jacky L. Snoep , Hans V. Westerhoff and Barbara M. Bakker

Supplementary Figure S1. Related to Figure 1 and 2

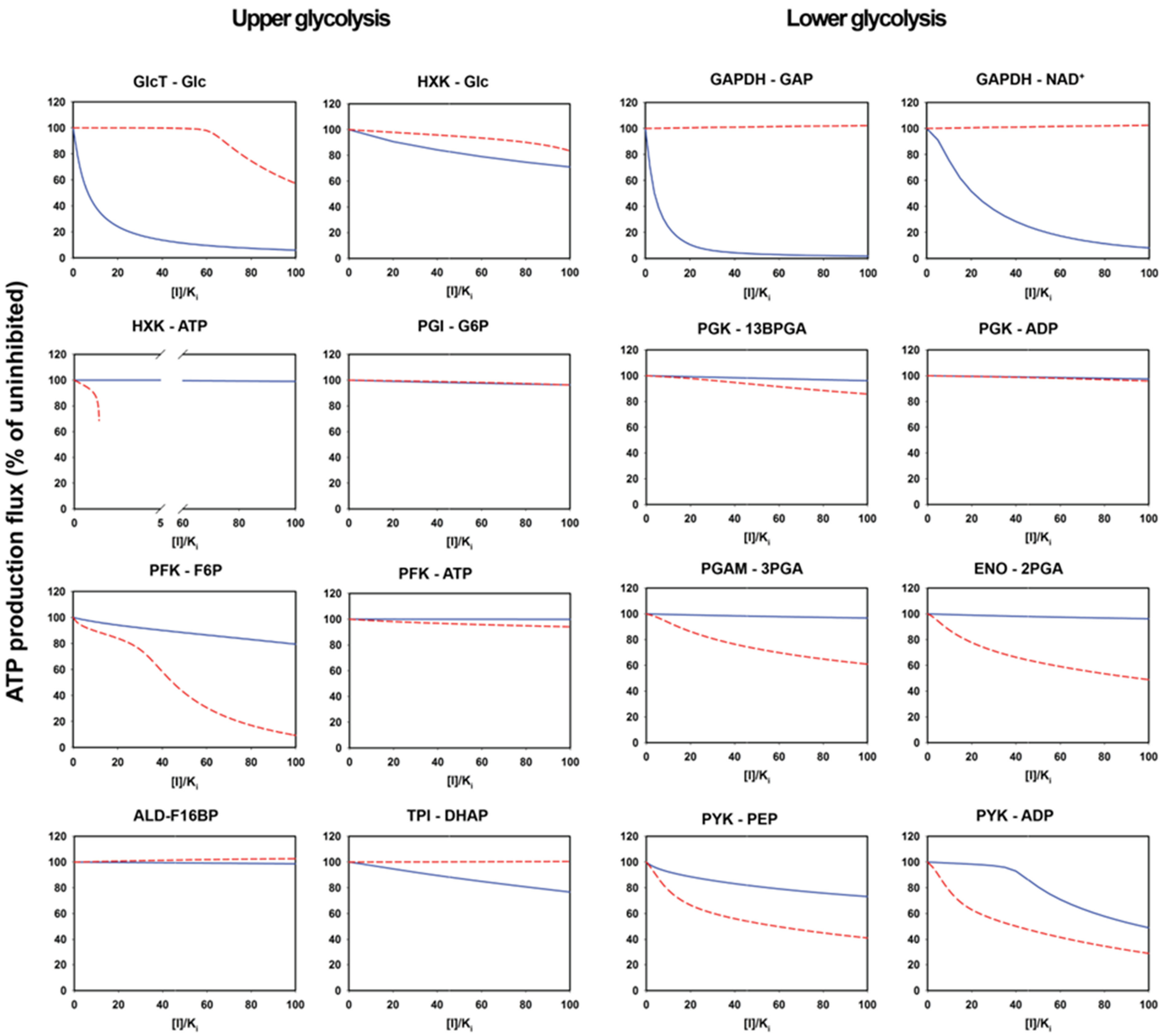

Competitive inhibition was simulated in the *T. brucei* glycolysis model (blue, solid line) and the erythrocyte glycolysis model (red, dashed line). Inhibition was titrated through increasing [inhibitor]/(inhibition constant of the inhibitor) ([I]/K_i). Using the [I]/K_i rather than the inhibitor concentration, generalizes the analysis to any inhibitor, irrespective of differences in potency at the molecular level. At each ([I]/K_i value, the steady state flux of the ATPase reaction was calculated which equals the ATP production flux and is plotted compared to the uninhibited flux. The target of the inhibitor and the competing substrate is indicated above each panel. For HXK, the inhibitor affected both the cytosolic and the glycosomal fraction in the *T. brucei* model. When a HXK inhibitor competitive with ATP was simulated, erythrocyte steady states could only be calculated up to an [I]/K_i of 1.4. The simulations were run up to 90% inhibition for the trypanosome model and this [I]/K_i-value was used to rank the target in Fig 1D. In a few cases the trypanosome model did not reach a steady state beyond a certain [I]/K_i. Then the highest [I]/Ki for which a steady state could be calculated was plotted in Fig 1D. This was the case for PFK- F6P (max [I]/K_i = 248) , PFK-ATP (max [I]/K_i = 507) and PGAM-3PGA (max [I]/K_i = 1.1x10^6). Note in the comparison with Fig 1D that the shape of the curve in the [I]/K_i range displayed here (0-100) may change at higher doses. For instance, the trypanosome curve for PYK-PEP decreases very slowly beyond [I]/K_i = 100, explaining why it ranks unfavorably in Fig 1D despite the initial flux decline shown here. GlcT: glucose transport, HXK:

hexokinase, PGI: phosphoglucoisomerase, PFK: phosphofructokinase, ALD: aldolase, TPI: triose phosphate isomerase, Glc: glucose, G6P: glucose-6-phosphate, F6P: fructose-6-phosphate, F16BP: fructose 1,6 bisphosphate, DHAP: dihydroxyacetone phosphate GAPDH: glyceraldehyde 3-phosphate dehydrogenase, PGK: phosphoglycerate kinase, PGAM: phosphoglycerate mutase, ENO: enolase, PYK: pyruvate kinase, GAP: glyceraldehyde 3-phosphate, 13BPGA: 1,3-bisphosphoglycerate, 3PGA: 3-phosphoglycerate, 2PGA: 2-phosphoglycerate, PEP: phospho*enol*pyruvate

Supplementary Figure S2 Related to Figure 3

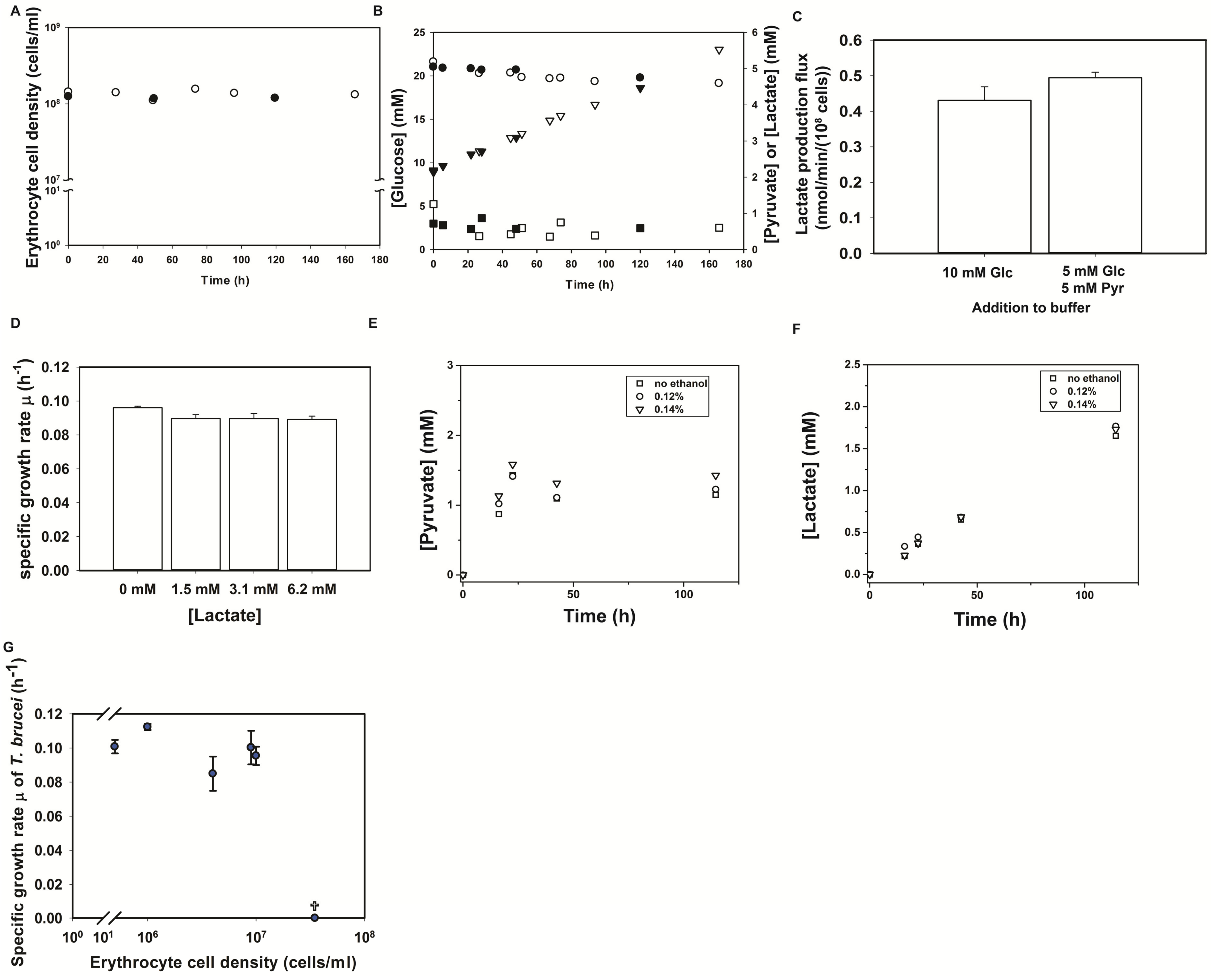

(A and B): Two independent batches of erythrocytes (black or white symbols) were washed in HEPES buffer, inoculated in in trypanosome cultivation medium (HMI-9) and followed for 5 or 7 days. (A) Cell densities of erythrocytes. Each data-point represents an averages of two counts (B) Metabolite concentrations in samples from the cultures as determined by HPLC as described previously [1]. Metabolites were separated on a Shimadzu HPLC with a Phenomenex column (Rezex ROA Organic Acid) and glucose was detected by a refractive index detector (RID-10A) and pyruvate and lactate by a UV detector (Spd-10A). Circles: glucose, triangles: lactate; squares: pyruvate. (C) Lactate production was followed by HPLC (see panel B) over 46h in monocultures of erythrocytes ($2\text{-}4\times10^{8}$ cells/ml) in HEPES-based buffer supplemented with 10 mM glucose (Glc) or 5 mM Glc and 5 mM pyruvate (Pyr). In the presence of pyruvate, the pyruvate consumption flux was 0.050±0.001 nmol/min/(10^{8} cells)). Error bar shows standard deviation of two independent cultures. (D) *T. brucei* was cultured in the presence of L-lactic acid (starting density 3×10^{5} trypanosomes/ml). Cell density was determined 4 times over a period of 27 hours and specific growth rate was calculated. Error bars show the error bars of two independent incubations. (E and F) Metabolite concentrations in co-cultures that were not treated at all, or were treated with ~0.1% ethanol (final concentration). Ratio of trypanosomes:erythrocytes is $1{:}10^{3}$. (G) Co-cultures of *T. brucei* (start density $1\text{-}5\times10^{5}$ cells/ml) and erythrocytes (density in graph) were counted 4-5 times over a period of 24-28 hours. The specific growth rate of *T. brucei* was calculated from these time-points as described previously [2]. The cross indicates that no *T. brucei* cells survived after 17 hours . Erythrocyte density stayed constant during the experiments. Error bars show standard deviation for 2-4 independent co-cultures.

Supplementary Figure S3. Related to Figure 4 and 5

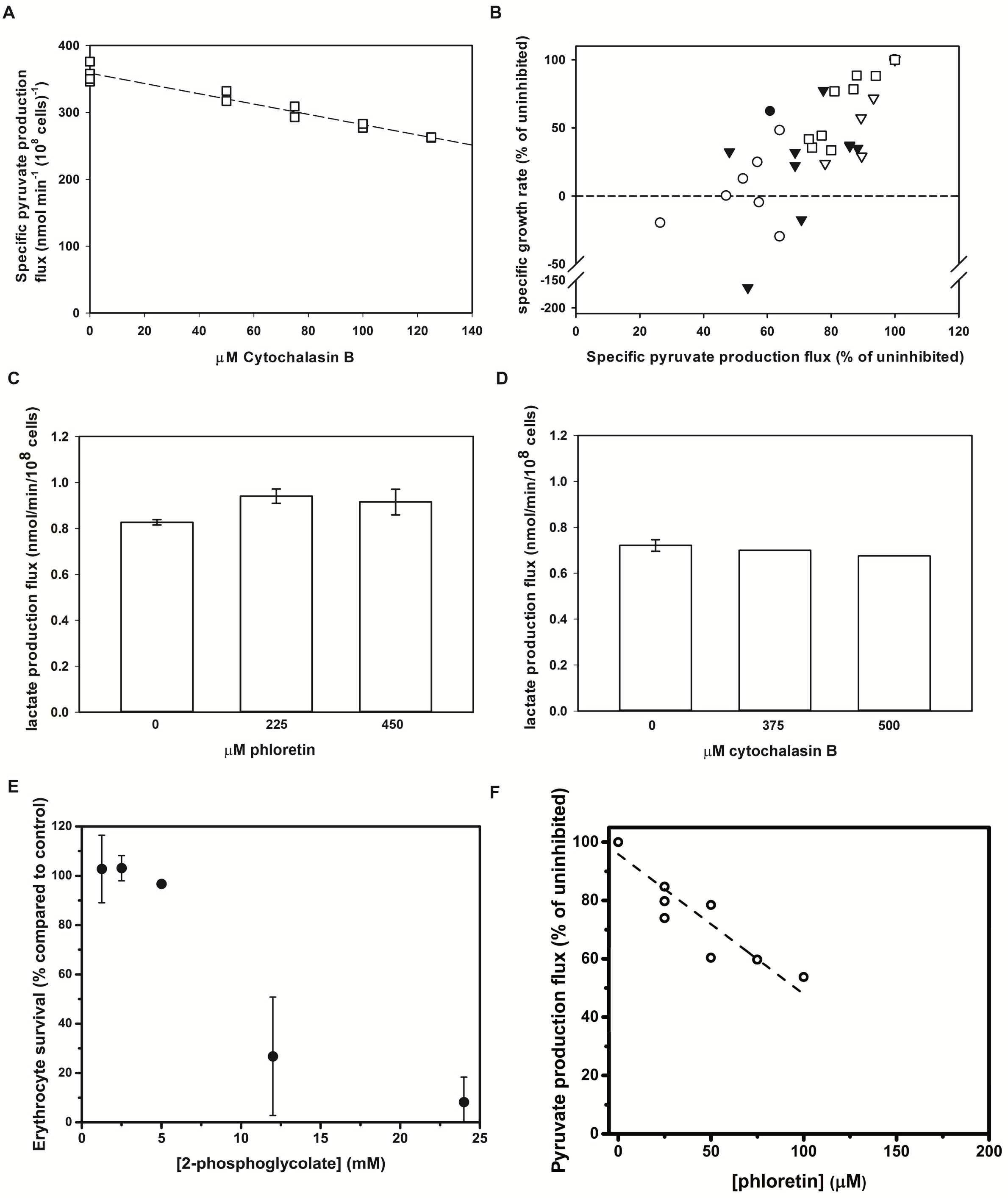

(A) Glycolytic flux (measured as the specific pyruvate production flux; based HPLC measurements on metabolite samples from cultures (see legend to Fig S2)) in bloodstream-form trypanosomes during 25h exposure to various concentrations of cytochalasin B. Each point in the graph is based on an independent culture. The dashed line is a linear fit through the datapoints (R^2 = 0.95). (B) Specific pyruvate production flux and growth rate in the cultures also shown in panel A were calculated as a percentage of uninhibited and plotted in the graph published previously in [2]. The squares are the results for cytochalasin B. Other results are for phloretin (black triangles), RNAi against various glycolytic enzymes (open circles) and a knockout of the alternative oxidase (black circles). (C and D):

Lactate concentration was measured in incubations of 0.8-1x10^8 erythrocytes/ml in HMI-9 over 90 70-90 hours in the presence of the indicated concentration inhibitor (C: Phloretin, D: Cytochalasin B) or solvent (control). Lactate was measured by HPLC as described in the legend to Fig S2. The flux calculation is based on 4-5 timepoints. Experiments with error bars (standard deviation) are done in duplicate. Co-cultures were for phloretin experiments with ≤0.4% v/v ethanol. Only in the cultures that were treated with cytochalasin B and their controls a concentration of 1.4% v/v ethanol was used due to the maximal solubility of cytochalasin B. (E) Aliquots of blood were washed twice in excess HEPES buffer without glucose and cells were resuspended in HEPES buffer containing 10 mM glucose at a concentration of ~2x10^7 cells/ml in a closed Eppendorf tube at 37°C. 2-phoshoglycolate (Sigma) was dissolved in DMSO and added to the erythrocyte suspension. Cell numbers were determined after 1 day (between 20-32 hours) in a Bürker haemocytometer and compared to DMSO-only controls. Shown is the mean with the standard deviation for 2 (2.5,5 mM and 12 mM), 3 (1.25 mM) or 4 (24 mM) independent incubations. (F) Same data as in Fig. 4E of the main text, but now without the 200 µM point and without the outlier at 100 µM. The linear fit has an R^2=0.86.

Supplementary Figure S4: Related to Figure 2

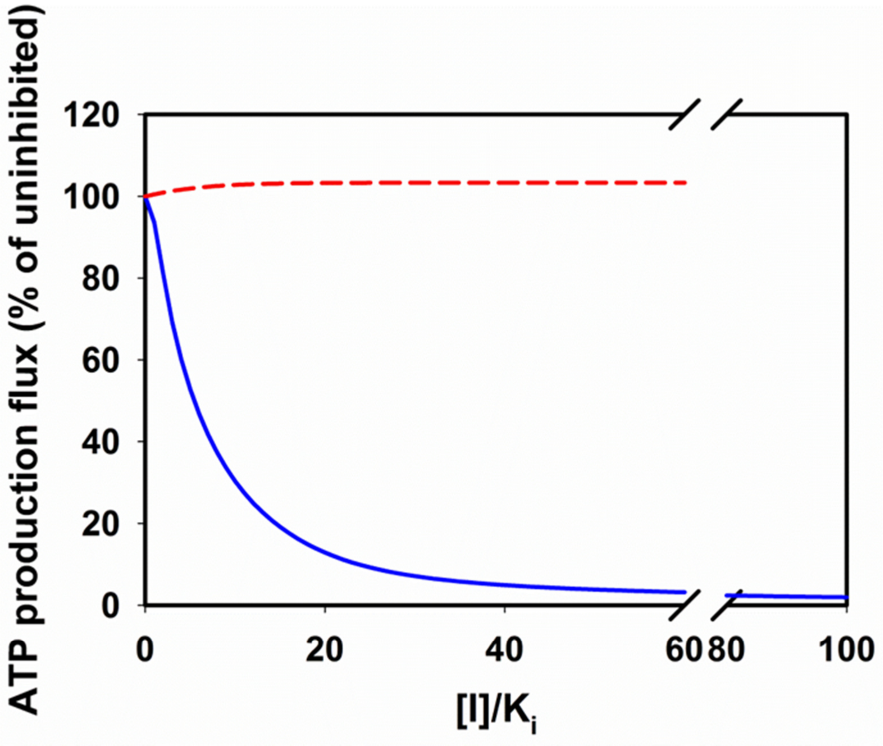

Uncompetitive inhibition of aldolase was modelled in each model by dividing both the V_{max} and the K_m values for Fru16BP and DHAP by 1 + $[I]/K_i$ [3]. Inhibition was simulated by titrating $[I]/K_i$ .Blue lines are the *T. brucei* model and the dashed red line is erythrocyte model. In the erythrocyte model, a steady state could be calculated up to an $[I]/K_i$ of 62.

Supplementary Figure S5. Related to Figure 5

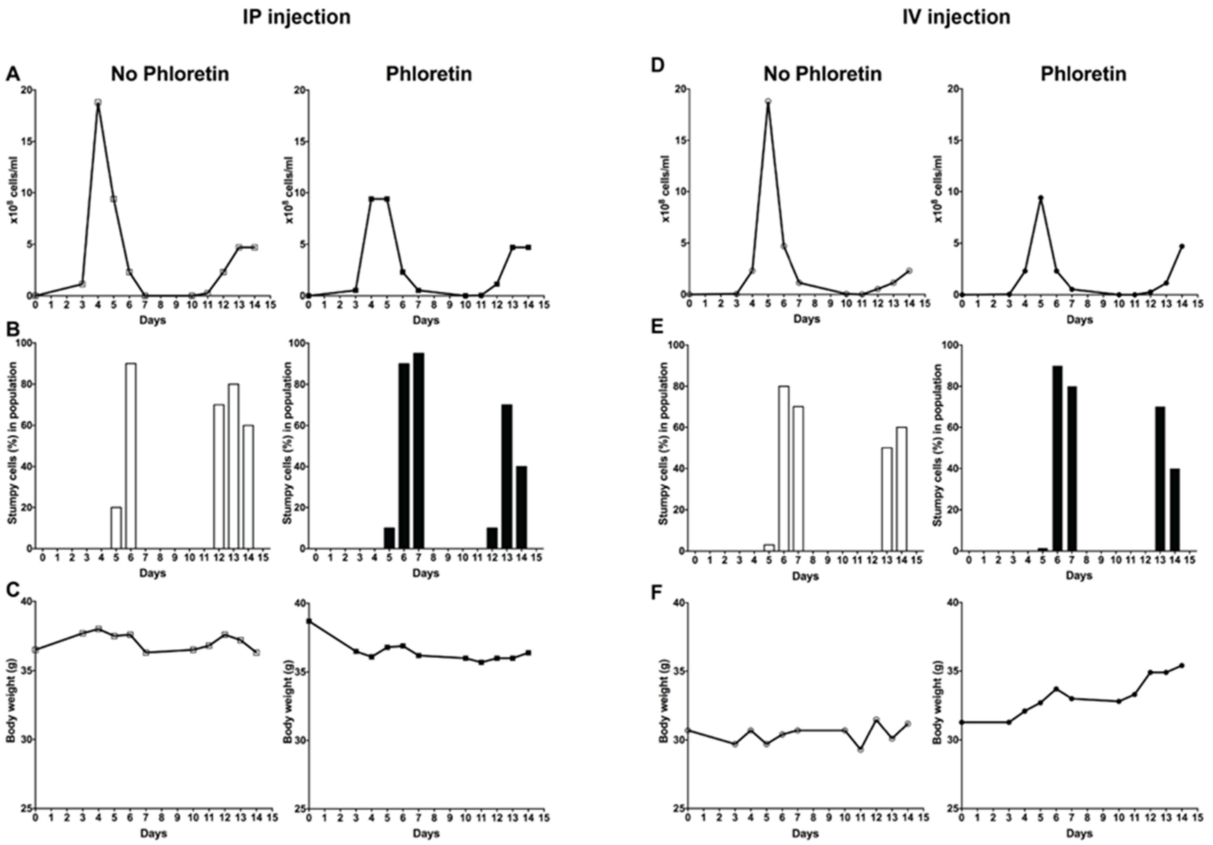

A. Graph shows the parasitaemia of individual mice through the course of infection with a parasite line (*T. brucei* AnTat1.1, see Extended Experimental methods below) B. shows the % of stumpy cells in the population (these being the parasite's transmission stage, generated at peak parasitaemia) and C. shows the weight changes of the mice throughout the experiment. The mice were injected intraperitoneally (with either DMSO control or phloretin) when trypanosomes were first detected in the blood sample (3rd day of infection). The first injection was followed by additional intraperitoneal injections on day 5, 7, 10 and 12. Results for all phloretin-treated mice were similar; here we show a representative result from the control group and the group treated with 10 mg/kg phloretin. D. Graph shows the parasitaemia through the course of infection with a parasite line (*T. brucei* AnTat1.1) able to generate chronic infections in vivo, E. shows the % of stumpy cells in the population (these being the parasite's transmission stage, generated at peak parasitaemia) and F. shows the weight changes of the mice throughout the experiment. Mice were injected intravenously (with either DMSO control or phloretin), when trypanosomes were first detected in the blood sample. (4th day of infection). This first injection was not followed by additional intravenous or intraperitonial injections. Results show the control mouse and the mouse injected with 10 mg/kg phloretin. The mouse injected with 50 mg/kg had to be sacrificed due to adverse side-effects.

Supplementary Figure S6. Related to Figure 6

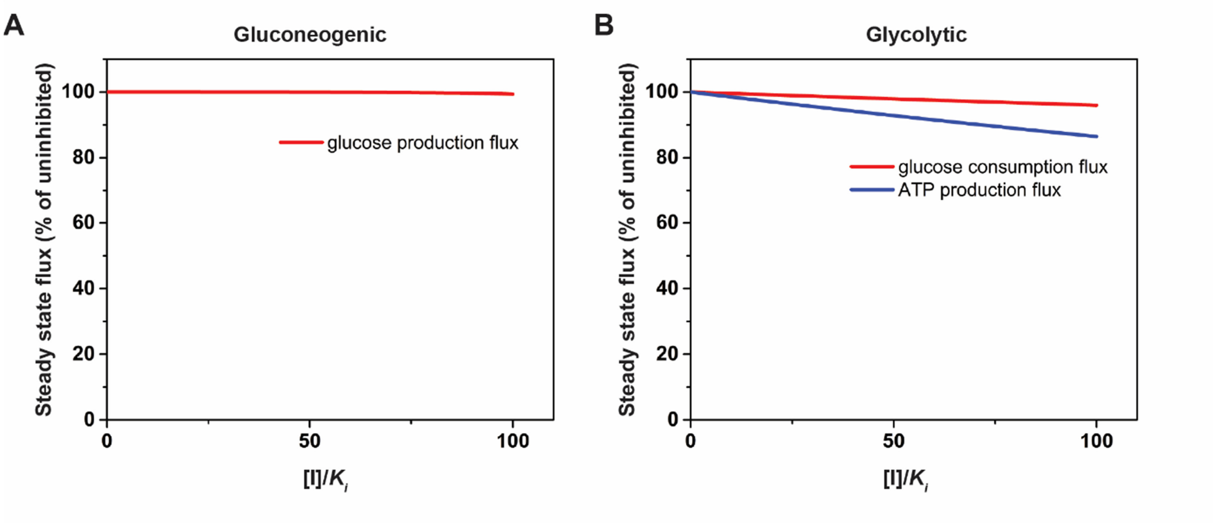

We used the hepatocyte glycolysis model from [4] in the same COPASI version as the *T. brucei* and erythrocyte glycolysis model (see main text methods). The model was evaluated in the steady state mode. To include competitive inhibition of glucose transport, we multiplied the K_m-values for the substrate (glucose in the blood) and the product (intracellular glucose) with the term (1 + ([I]/K_i) and titrated [I]/K_i from 0-100. (A) Simulations at increasing [I]/K_i under gluconeogenic conditions (0 mM glycogen, 1 mM blood glucose, 1.2 mM blood lactate). Under these conditions the liver cells predominantly excrete glucose to the blood which they make through gluconeogenesis from the lactate (B) Simulations at increasing [I]/K_i under glycolytic conditions (5 mM glycogen, 7 mM blood glucose, 0 mM blood lactate). In this case, the hepatocytes take up glucose from the blood and produce ATP through glycolysis. The flux through pyruvate kinase was plotted as a measure of net ATP production.

Supplementary Figure S7. Related to Figure 6

phloretin

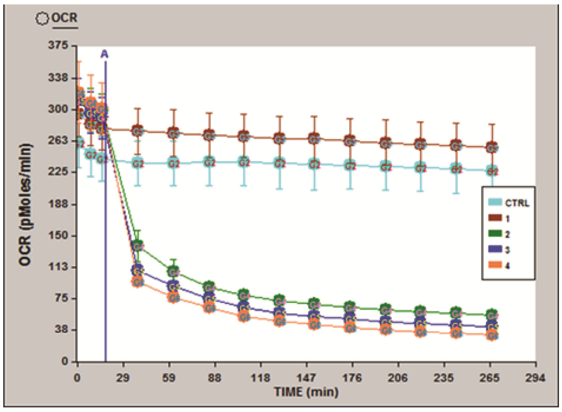

cytochalasin B

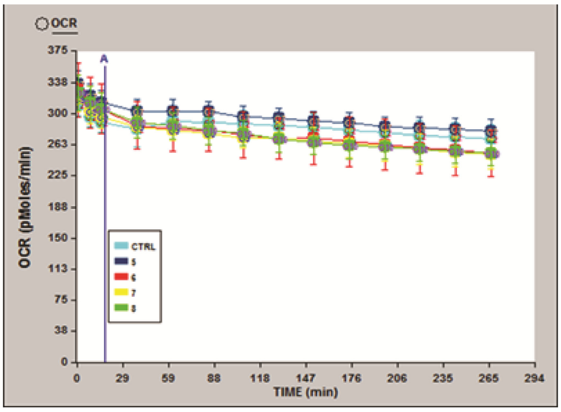

Oxygen consumption rate (OCR) measurements for HT-22 cells treated with phloretin or cytochalasin B. For phloretin: Ctrl = nothing added, 1 = ethanol control , 2= 50 µM, 3 = 100 µM, 4 = 125 µM. For Cytochalasin B: Ctrl = nothing added, 5 = ethanol control, 6= 100 µM, 7 = 150 µM, 8 = 250 µM. Shown here are the results of one biological replicate for each inhibitor concentration. For phloretin the error bars denote the standard deviation of 6 technical replicates, for cytochalasin B the error bars denote the standard deviation of 3 technical replicates.

Supplementary Table S1. Related to Figure 2 and methods

The K_m values listed in this table were multiplied simultaneously by a factor 1 + [I]/K_i in order to simulate the binding of a competitive inhibitor to the active site of the enzyme.

Enzyme<br>competing metabolite	***T. brucei****	**Erythrocyte****
Glc Transport***<br>Glc_{out}	$K_{m,Glc}$; α	$K_{m,in}$; $K_{m,out}$; α
HXK<br>Glc_{in}<br>ATP	 $K_{m,Glc}$; $K_{m,Glc6P}$<br>$K_{m,ATP}$; $K_{m,ADP}$	 K_{Glc}<br>K_{MgATP}; $K_{MgATP,Mg}$
PGI<br>Glc6P	 $K_{m,Glc6P}$; $K_{m,Fru6P}$	 K_{Glc6P}; K_{Fru6P}
PFK<br>Fru6P<br>ATP	 $K_{m,Fru6P}$; K_{i2}<br>$K_{m,ATP}$, $K_{m,ADP}$	 K_{Fru6P}<br>K_{MgATP}
ALD<br>Fru16BP	 $K_{m,Fru16BP}$; $K_{m,DHAP}$	 $K_{Fru1,6P2}$; K_{DHAP}
TPI<br>DHAP	 $K_{m,DHAP}$; $K_{m,GA3P}$	 K_{DHAP}; K_{GraP}
GAPDH<br>GA3P<br>NAD^+	 $K_{m, GA3P}$; $K_{m, 13BPGA}$<br>$K_{m, NAD+}$; $K_{m, NADH}$	 K_{GraP}; $K_{1,3P2G}$<br>K_{NAD}; K_{NADH}
PGK<br>13BPGA<br>ADP	 $K_{m, 13BPGA}$; $K_{m, 3PGA}$<br>$K_{m,ATP}$; $K_{m,ADP}$	 $K_{1,3P2G}$; K_{3PG}<br>K_{MgADP}; K_{MgATP}
PGAM<br>3PGA	$K_{m,3PGA}$;$K_{m,2PGA}$	K_{3PG};K_{2PG}
ENO<br>2PGA	$K_{m,2PGA}$; $K_{m,PEP}$	K_{2PG}; K_{PEP}
PYK<br>PEP<br>ADP	 $K_{m,PEP;}$ $K_{m,pyruvate}$<br>$K_{m,ADP;}$ $K_{m,ATP}$	 K_{PEP}<br>K_{MgADP}

* Nomenclature according to ref [5]

** Nomenclature according to the Supplementary Material to [6]. The equilibrium constant was included explicitly in each rate equation, so that the inhibition kinetics could not shift the equilibrium.

*** Glucose transport occurs via facilitated diffusion and hence the equation for the glucose transporter is different from the classical Michaelis-Menten equations. We have previously derived how a competitive inhibitor should affect the transport step (see supplement to [7]): a competitive inhibitor would simultaneously affect the K_m and the α. The extent to which an inhibitor affects these parameters depends on the ratio of two inhibition constants K_{i1} and K_{i2}. The ratio K_{i2}/K_{i1} can theoretically range between 0.5 and infinity. The simulations here consider the case that this ratio is infinity.

Supplementary Table S2. Related to Figure 2

		Flux control coefficient C_i^J			elasticity coefficient $*\,\varepsilon^i_{[I]/K_i}$			$(C_i^J)\cdot(*\,\varepsilon^i_{[I]/K_i})$		
enzyme *i*	competing substrate	*T. brucei*	erythrocyte	ratio *T. brucei*/erythrocyte	*T. brucei*	erythrocyte	ratio *T. brucei*/erythrocyte	*T. brucei*	erythrocyte	ratio *T. brucei*/erythrocyte
**GlcT**	glucose	6.0×10^{-1}	1.0×10^{-4}	5.8×10^{3}	-1.6×10^{-1}	-1.8×10^{-1}	9.0×10^{-1}	-9.6×10^{-2}	-1.8×10^{-5}	5.3×10^{3}
**HXK***	glucose	3.3×10^{-2}	5.2×10^{-2}	6.4×10^{-1}	-8.1×10^{-1}	-2.1×10^{-2}	3.8×10^{1}	-2.7×10^{-2}	-1.1×10^{-3}	2.4×10^{1}
	ATP	3.3×10^{-2}	5.2×10^{-2}	6.4×10^{-1}	-2.0×10^{-2}	-1.4	1.4×10^{-2}	-6.6×10^{-4}	-7.3×10^{-2}	9.0×10^{-3}
**PGI**	G6P	1.3×10^{-3}	3.5×10^{-4}	3.9	-3.6×10^{-1}	-7.0×10^{-1}	5.2×10^{-1}	-4.9×10^{-4}	-2.4×10^{-4}	2.0
**PFK**	F6P	5.3×10^{-3}	2.6×10^{-2}	2.0×10^{-1}	-8.6×10^{-1}	-1.4	6.3×10^{-1}	-4.5×10^{-3}	-3.6×10^{-2}	1.3×10^{-1}
	ATP	5.3×10^{-3}	2.6×10^{-2}	2.0×10^{-1}	-2.0×10^{-3}	-4.6×10^{2}	4.4×10^{-2}	-1.1×10^{-5}	-1.2×10^{-3}	8.9×10^{-3}
**ALD**	F16BP	4.2×10^{-2}	-5.7×10^{-3}	-7.4	-3.0×10^{-3}	-6.7×10^{-2}	4.5×10^{-2}	-1.3×10^{-4}	3.8×10^{-4}	-3.3×10^{-1}
**TPI**	DHAP	8.9×10^{-3}	-5.6×10^{-5}	-1.6×10^{2}	-2.7×10^{-1}	-8.4×10^{-1}	3.2×10^{-1}	-2.4×10^{-3}	4.7×10^{-5}	-5.2×10^{1}
**GAPDH**	GAP	1.5×10^{-1}	-6.2×10^{-4}	-2.4×10^{2}	-5.3×10^{-1}	-4.4×10^{-1}	1.2	-7.8×10^{-2}	2.8×10^{-4}	-2.8×10^{2}
	NAD	1.5×10^{-1}	-6.2×10^{-4}	-2.4×10^{2}	-8.0×10^{-2}	-4.4×10^{-1}	1.8×10^{-1}	-1.2×10^{-2}	2.8×10^{-4}	-4.3×10^{1}
**PGK**	13BPGA	7.4×10^{-3}	9.7×10^{-4}	7.6	-5.0×10^{-2}	-8.9×10^{-1}	5.6×10^{-2}	-3.7×10^{-4}	-8.6×10^{-4}	4.3×10^{-1}
	ADP	7.4×10^{-3}	9.7×10^{-4}	7.6	-3.0×10^{-2}	-2.6×10^{-1}	1.1×10^{-1}	-2.2×10^{-4}	-2.6×10^{-4}	8.7×10^{-1}
**PGAM**	3PGA	6.2×10^{-2}	4.2×10^{-3}	1.5×10^{1}	-7.0×10^{-3}	-9.8×10^{-1}	7.2×10^{-3}	-4.3×10^{-4}	-4.1×10^{-3}	1.0×10^{-1}
**ENO**	2PGA	1.1×10^{-2}	7.8×10^{-3}	1.4	-5.0×10^{-2}	-9.8×10^{-1}	5.1×10^{-2}	-5.6×10^{-4}	-7.6×10^{-3}	7.4×10^{-2}
**PYK**	PEP	8.3×10^{-3}	2.4×10^{-2}	3.5×10^{-1}	-1.9	-1.0	1.8	-1.6×10^{-2}	-2.5×10^{-2}	6.4×10^{-1}
	ADP	8.3×10^{-3}	2.4×10^{-2}	3.5×10^{-1}	-1.1×10^{-1}	-7.8×10^{-1}	1.4×10^{-1}	-9.2×10^{-4}	-1.8×10^{-2}	5.0×10^{-2}

* For *T. brucei* only the glycosomal hexokinase was considered in this analysis

Ratios were calculated before numbers were rounded off.

Colour-coding ratios (absolute values): green: $\geq 10^{2}$ (selectivity towards *T. brucei*); red: $\leq 10^{-2}$ (selectivity towards erythrocyte), yellow: all others (no strong selectivity to either cell types)

Supplementary Table S3: Related to Figure 5 and S5

Phloretin concentrations in blood samples were determined with a HPLC-based method described in [8].

Sample	ratio phloretin/diadzein	ug	ng/ml	uM
Mouse 1; control; 1hr post-injection	0.002	0.000912	36	0.13
Mouse 2; 10 mg/kg ; 1 hr post-injection	0.001	0.000541	22	0.08
Mouse 3; 50 mg/kg ; 1 hr post-injection	0.001	0.000393	16	0.06
Mouse 1; control; 24hr post-injection	0.000	0.000283	14	0.05
Mouse 2; 10 mg/kg ; 24 hr post-injection	0.000	0.000245	12	0.04

Supplementary methods

Simulation of inhibition kinetics

Competitive inhibition was modelled by multiplying the relevant K_m values by the factor $(1 + [I]/K_i)$ [3]. The K_m of the substrate as well as that of the corresponding product was modified by the same factor, since substrate, product and inhibitor all compete for the active site. The only exception is when the product inhibits through binding to an allosteric site, such as allosteric inhibition of erythrocyte HXK by Glc6P. In the latter case, only the K_m for the substrate was modified, while the K_m for the product was unaffected by the inhibitor. In all cases the equilibrium constant was unaffected by the inhibitors. Table S1 lists the K_m values that were changed in each particular case.

Calculation of coefficients

The flux control coefficient C_i^J of an enzyme i over a steady-state flux J is defined as:

$$C_i^J = \frac{d\ln J/dp}{\partial \ln v_i/\partial p} \quad \text{(Eq. 8)}$$

in which v_i is the rate of enzyme *i* and *p* is any parameter that only affects v_i [9]. Since in each of the reactions considered v_i was proportional to the V_{max} of the enzyme, the V_{max} was the parameter of choice. Flux control coefficients were calculated via the metabolic control analysis function in COPASI. We checked that all flux control coefficients over the ATP utilization flux added up to 1.

The partially normalized elasticity coefficient towards the $[I]/K_i$ ratio represents the direct effect of the inhibitor on the enzyme rate v_i and was calculated at zero [I] according to the definition:

$$^*\varepsilon^i_{[I]/K_i} = \frac{\partial v_i/v_i}{\partial([I]/K_i)} \quad \text{(Eq. 9)}$$

The asterisk indicates that this elasticity coefficient deviates from the standard definition [9], which would normalize for the actual concentration of the inhibitor. This modified elasticity coefficient is independent of the actual value of the inhibition constant and also exists in the limit $[I]\rightarrow 0$.

The partially normalized elasticity coefficient of an enzyme *i* towards its inhibitor ($^*\varepsilon^i_{[I]/K_i}$) was calculated as follows: The derivative of the enzyme rate v_i with respect to $[I]/K_i$ was determined symbolically using Mathematica 9 software and the outcome was calculated at $[I]/K_i = 0$ and at the steady-state metabolite concentrations in the absence of inhibitor. The resulting value was divided by the steady-state enzyme rate to obtain $^*\varepsilon^i_{[I]/K_i}$.

More on the Measurements of Oxygen Consumption Rate (OCR) and Extracellular acidification rate (ECAR)

HT-22 cells were seeded in XF 96-well cell culture microplates (Seahorse Bioscience) at a density of 10,000 cells/well in 4.5 g/liter of standard culture medium and incubated at 37°C and 5% CO2 for ~24 hours. Before starting the measurements, the growth medium was washed away and replaced with ~180 µl of assay medium (with 4.5 g/liter of glucose (25 mM) as the sugar source, 2 mM glutamine, 1 mM pyruvate, pH 7.35), and cells were incubated at 37°C for at least 60 minutes. Three baseline measurements were recorded before the addition of compounds. Phloretin, cytochalasin B and ethanol-vehicle control were added in Port A at different concentrations and OCR/ECAR values were continuously monitored for 4 hours. To assess mitochondrial function further, respiratory chain inhibitors were used. The ATP synthase inhibitor oligomycin was injected through port B at a final concentration of 3 µM to measure OCR in the absence of oxidative phosphorylation. The protonophore carbonylcyanide-4-(trifluormethoxy)-phenylhydrazone was subsequently injected in port C at a concentration of 0.4 µM to dissipate the proton gradient across the inner mitochondrial membrane and thereby to assess mitochondrial respiratory capacity (MRC). Co-injection of the

complex I/III inhibitors rotenone/antimycin A together with 2-DG in port D at concentrations of 1 µM and 500 µM respectively was used to completely inhibit O2 consumption and glycolysis. Three measurements were performed after the addition of each compound by a 4-minute mix cycle used to oxygenate the medium and a 3-minute measurement cycle to assess respiration. For comparing OCR after compound exposure to OCR in non-treated cells, the absolute cell number is insignificant as a result of the same population of cells being compared. Therefore, results are indicated as normalized OCR (% baseline rate) for each individual cell population to minimize variability resulting from slight differences in plating and viability during culture and treatment time (~5 hours).

Enrichment of erythrocytes containing plasmodium falciparum *in trophozoite stage*

Trophozoite-stage packed erythrocytes (pRBCs) were enriched using a density gradient separation protocol using layers of 60% and 85% Percoll in RPMI, glucose and HEPES, pH 7.2. [10]. Alanine (3%, final concentration) was added to the mixture of each layer. On top of the Percoll layers, 200-250 uL of packed pRBC's (collected at 750xg, 3 min) was added and centrifuged immediately (9600xg, 20 min; zero deceleration). The layer between the 85% and 60% Percoll layers, which contained the enriched trophozoite-stage pRBCs was washed with pre-warmed (37°C) 5x phosphate buffered saline (PBS) and spun (600xg, 3 min). The cells were resuspended and incubated for 30 min at 37°C to allow the remaining alanine to equilibrate. The suspension was spun again (600xg, 5 min) to produce a pellet of infected cells that was used for further experiments.

Glycolytic flux measurements and inhibition for plasmodium-infected erythrocytes

The metabolic activity of infected-RBCs was determined by incubating enriched (near 100% infected), intact pRBCs in RPMI-1640 and HEPES (50 mM) buffer with 5 mM glucose and measuring enzymatically the lactate produced over 1 hr (5 time points) in absence and presence of cytochalasin B (dissolved in DMSO, final concentration < 0.5% m/v; control received only DMSO). Cell numbers were determined in a Neubauer haemocytometer and exclusion of 0.04% v/v trypan blue confirmed cell integrity. Time point samples were collected and centrifuged (800xg, 1 min) and the supernatant kept for the enzymatic determination of extracellular lactate using an LDH and NAD^+ linked assay. Standards and samples were incubated in a HEPES assay buffer (150 mM HEPES; pH 7.6) containing L-LDH (11 U/mL), NAD^+ (1.6 mM), hydrazine (16 µL/mL) and incubated for 90 mins at 21 °C. Absorbance was measured at 340 nm (Varioscan microplate reader Thermo Electron Corporation Waltham, Massachusetts, USA).

Animals and pleoiomorphic trypanosomes

Animal experiments were carried out according to the UK Animals (Scientific) Procedures act under a licence (60/4373) issued by the UK Home Office and approved by the University of Edinburgh Local ethical committee. As this was a pilot study, the sample size was small and there was no randomisation or blinding done

T. brucei AnTat1.1 (approximately $5x10^5$ parasites) were infected into age-matched MF1 female mice. Parasitaemia was monitored by microscopy of bloodsmears every day through the 14 days of the experiment by counting trypanosomes and extrapolating to the actual cells/ml [11]. This pleomorphic strain is as sensitive to phloretin as the Lister 427 strain that we used in the in vitro experiments [12]. Phloretin has in the past been used to treat HepG2 adenocarcinomas in mice [13] with no reported side effects, making it the compound of choice for in vivo testing.

In the experiments a fresh batch of phloretin was used (Sigma-Aldrich), after the killing effect of the drug was established on AnTat1.1s in culture.

Intraperitonial phloretin injections

Three mice groups were set up, two mice in each group.

The mice in these three groups were injected intraperitoneally (with either DMSO control or phloretin) when trypanosomes were first detected in the blood sample (3rd day of infection). The first injection was followed by additional intraperitoneal injections on day 5, 7, 10 and 12. The mice were injected intraperitoneally with 200 µl solution with either 20% DMSO (group 1), a phloretin dose in 10% DMSO that would lead to administration of 10 mg/kg phloretin (group 2), or a phloretin dose in 20% DMSO that would lead to administration of 25 mg/kg phloretin (goup 3)

Intravenous phloretin injections

Three mice were injected intravenously (with either DMSO control or phloretin), when trypanosomes were first detected in the blood sample (4th day of infection). This first injection was not followed by additional intravenous or intraperitonial injections. The mice were injected intraperitoneally with 200 µl solution with either 20% DMSO, with a phloretin dose in 20% DMSO that would lead to administration of 10 mg/kg or 50 mg/kg phloretin. A blood sample was taken from each mouse 1 and 24h post-injection.

Considering an average mouse weight of 25 gram, a total blood volume for a mouse of 1.5 mL (https://www.nc3rs.org.uk/mouse-decision-tree-blood-sampling) and the molecular weight of phloretin of 274 gram/mole, a 10 mg/kg injection of phloretin would amount to a 600 µM concentration if it remains in the blood and a 36 µM concentration if the phloretin would get dispersed equally over the entire mouse.

Supplementary information references

1. Haanstra JR*, et al.* Proliferating bloodstream-form *Trypanosoma brucei* use a negligible part of consumed glucose for anabolic processes. *International journal for parasitology* **42**, 667-673 (2012).
2. Haanstra JR*, et al.* A domino effect in drug action: from metabolic assault towards parasite differentiation. *Mol Microbiol* **79**, 94-108 (2011).
3. Cornish-Bowden A. *Fundamentals of Enzyme Kinetics*, 3rd edn. Portland Press (2004).
4. Konig M, Bulik S, Holzhutter HG. Quantifying the contribution of the liver to glucose homeostasis: a detailed kinetic model of human hepatic glucose metabolism. *PLoS computational biology* **8**, e1002577 (2012).
5. Kerkhoven EJ*, et al.* Handling uncertainty in dynamic models: the pentose phosphate pathway in *Trypanosoma brucei*. *PLoS computational biology* **9**, e1003371 (2013).
6. Holzhutter HG. The principle of flux minimization and its application to estimate stationary fluxes in metabolic networks. *European journal of biochemistry / FEBS* **271**, 2905-2922 (2004).
7. Bakker BM*, et al.* Contribution of glucose transport to the control of the glycolytic flux in Trypanosoma brucei. *Proc Natl Acad Sci U S A* **96**, 10098-10103 (1999).
8. Remsberg CM, Yáñez JA, Vega-Villa KR, Miranda ND, Andrews PK, Davies NM. HPLC-UV Analysis of Phloretin in Biological Fluids and Application to Pre-Clinical Pharmacokinetic Studies. *Journal of Chromatography & Separation Techniques* **1**, (2010).
9. Heinrich R, Schuster S. *The regulation of cellular systems*. Chapmann and Hall (1996).
10. Kutner S, Breuer WV, Ginsburg H, Aley SB, Cabantchik ZI. Characterization of permeation pathways in the plasma membrane of human erythrocytes infected with early stages of Plasmodium falciparum: association with parasite development. *J Cell Physiol* **125**, 521-527 (1985).
11. Herbert WJ, Lumsden WH. Trypanosoma brucei: a rapid "matching" method for estimating the host's parasitemia. *Experimental parasitology* **40**, 427-431 (1976).
12. Szoor B, Dyer NA, Ruberto I, Acosta-Serrano A, Matthews KR. Independent pathways can transduce the life-cycle differentiation signal in Trypanosoma brucei. *PLoS pathogens* **9**, e1003689 (2013).
13. Wu CH*, et al.* In vitro and in vivo study of phloretin-induced apoptosis in human liver cancer cells involving inhibition of type II glucose transporter. *International journal of cancer Journal international du cancer* **124**, 2210-2219 (2009).
